# Bioinformatic analysis reveals new determinants of antigenic 14-3-3 proteins and a novel antifungal strategy

**DOI:** 10.1371/journal.pone.0189503

**Published:** 2017-12-12

**Authors:** Jenna E. McGowan, Jacqueline Kratch, Saurabh Chattopadhyay, Bina Joe, Heather R. Conti, Ritu Chakravarti

**Affiliations:** 1 Department of Surgery, University of Toledo College of Medicine & Life Sciences, Toledo, OH, United States of America; 2 Department of Biological Sciences, University of Toledo, Toledo, OH, United States of America; 3 Department of Medical Microbiology & Immunology, University of Toledo College of Medicine & Life Sciences, Toledo, OH, United States of America; 4 Department of Physiology & Pharmacology, University of Toledo College of Medicine & Life Sciences, Toledo, OH, United States of America; Louisiana State University, UNITED STATES

## Abstract

The ubiquitously expressed 14-3-3 family of proteins is evolutionarily conserved from yeast to mammals. Their involvement in humoral and cellular immune responses is emerging through studies in drosophila and humans. In humans, a select group of 14-3-3 isoforms are antigenic; however the determinants of their antigenicity are not known. Here, we show that although mammalian 14-3-3 proteins are mostly conserved, subtle differences between their isoforms may give rise to their antigenicity. We observed syntenic relations among all the isoforms of 14-3-3 for mammals, but not with that of birds or amphibians. However, the parasitic 14-3-3 isoforms, which have known antigenic properties, show unique sequence, structure and evolution compared to the human 14-3-3. Moreover we report, for the first time the existence of a bacterial 14-3-3 protein. Contrary to the parasitic isoforms, both bacterial and yeast 14-3-3 exhibited significant homology with mammalian 14-3-3 in protein sequence as well as structure. Furthermore, a human 14-3-3 inhibitor caused significant killing of *Candida albicans*, which could be due to the inhibition of the structurally similar yeast homologue of 14-3-3, BMH, which is essential for its life cycle. Overall, our bioinformatic analysis combined with the demonstration of a novel antifungal role of a peptide inhibitor of human 14-3-3 indicates that the sequences and structural similarities between the mammalian, bacterial and fungal proteins are likely determinants of the antigenic nature of these proteins. Further, we propose that molecular mimicry triggered by microbial infections with either yeast or bacteria may contribute to the antigenic role of human 14-3-3.

## Introduction

The 14-3-3 family of proteins is evolutionarily conserved in eukaryotes ranging from yeast to mammals. They are well-studied for their role as adaptor proteins in a multitude of signaling pathways leading to cellular proliferation, differentiation and death [1]. Dysregulation of function or expression of several isoforms has been reported in multiple human diseases [2–6]. Among these, increased expression of 14-3-3 has been observed in body fluids of patients with autoimmune diseases such as rheumatoid arthritis and multiple sclerosis, suggesting an immunological function for 14-3-3 proteins[7, 8]. This is also evident in other organisms, e.g. the loss of 14-3-3ε in Drosophila is linked with defects in exocytosis of immune mediators leading to enhanced susceptibility to bacterial infection [9, 10], 14-3-3ζ is associated with phagocytosis and resistance against *S*. *aureus* in both drosophila and zebrafish [11], and 14-3-3 proteins in plants regulate the pathogenic interactions [12]. These relatively new data are interesting to further investigate how 14-3-3 functions contribute to immune responses. In this regard, it is interesting to note that antigenic properties of 14-3-3 have recently been identified. Antibodies against 14-3-3 are reported in autoimmune, cancer and infectious disease [13–18]; 14-3-3η is antigenic in rheumatoid arthritis [15] and 14-3-3ζ is antigenic in cancer [14, 18]. We and others have reported the increased antigenicity of 14-3-3ζ and 14-3-3ε in aneurysmal inflamed thoracic aorta [13, 19]. In contrast to these observations, in aneurysmal aortitis, 3 isoforms (beta, tau and gamma) of 14-3-3 family that are found to be upregulated, are not antigenic, suggesting that antigenicity could be an independent function in some diseases or individual isoforms may have different roles in different diseases [13]. Therefore, more information is needed to better understand the factors dictating the antigenic properties of 14-3-3. This in turn may guide the development of specific diagnostic assays for 14-3-3 and its isoforms as clinical markers of autoimmune diseases. We approached this issue with a bioinformatic perspective to compare and contrast the evolutionary structure-function relationships of 14-3-3. There have been several reports of comparative analyses of 14-3-3 family proteins across the species [20–24], but our efforts are directed to specifically understand its new antigenic function.

Our results revealed high degree of structural and phylogenetic similarities between the human, yeast and bacterial 14-3-3 but not with other organisms such as protozoans. Some isoforms of 14-3-3 (including ζ and ε) are better known for their antigenic function [13–15, 18, 19]; therefore, we performed comparative analyses on these isoforms, primarily ζ. Thus, molecular mimicry triggered by microbial infections may contribute to the antigenic role of human 14-3-3. Furthermore, by assessing the functional similarity in humans and yeast, our study reports a novel antifungal role of a peptide inhibitor of human 14-3-3.

## Results

### Variability in highly conserved mammalian 14-3-3 proteins

**Fig 1:**
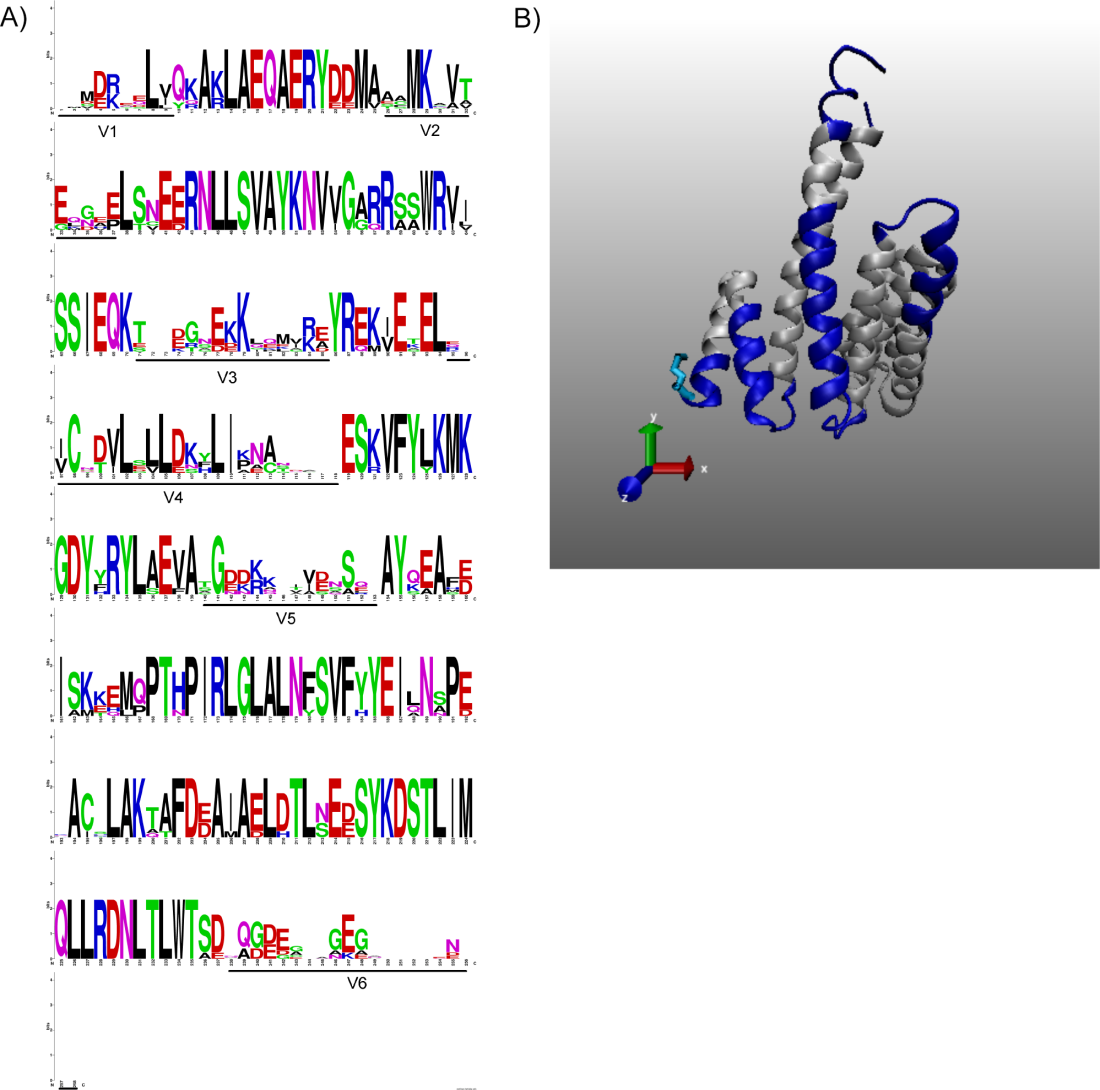
Bioinformatic analyses identified six variable regions in human 14-3-3 isoforms. Human 14-3-3 isoforms were aligned using the MUSCLE program and analyzed at WebLogo to identify the conservation of protein sequences. Areas of variability are underlined and are numbered as V1 to V6. (B) Structure of 14-3-3 with variable regions in solid blue color is shown. Human 14-3-3ζ (PDB ID 5D2D) was used as a template to overlay the variable domains. N-terminal methionine is indicated in light blue. Variable regions primarily located at the end of helices and in the unstructured regions.

The seven human isoforms of 14-3-3 (accession number in S1 Table) were aligned to identify regions of conservation and variation. Overall, there was up to 80% homology between the isoforms (S1 Fig). Logo analysis, which shows the highly conserved residues with a greater height [25], also revealed significant conservation among family; however, there were six distinct regions of variation (V1-V6, underlined in Fig 1A). The notable variability defined by ≤50% similarity of residues between proteins was confirmed by the Logo analysis and sequence alignment (S1 Fig). Structurally these variable regions corresponded to the unstructured regions at the ends of helices of the protein structure of 14-3-3 (Fig 1B).

**Fig 2:**
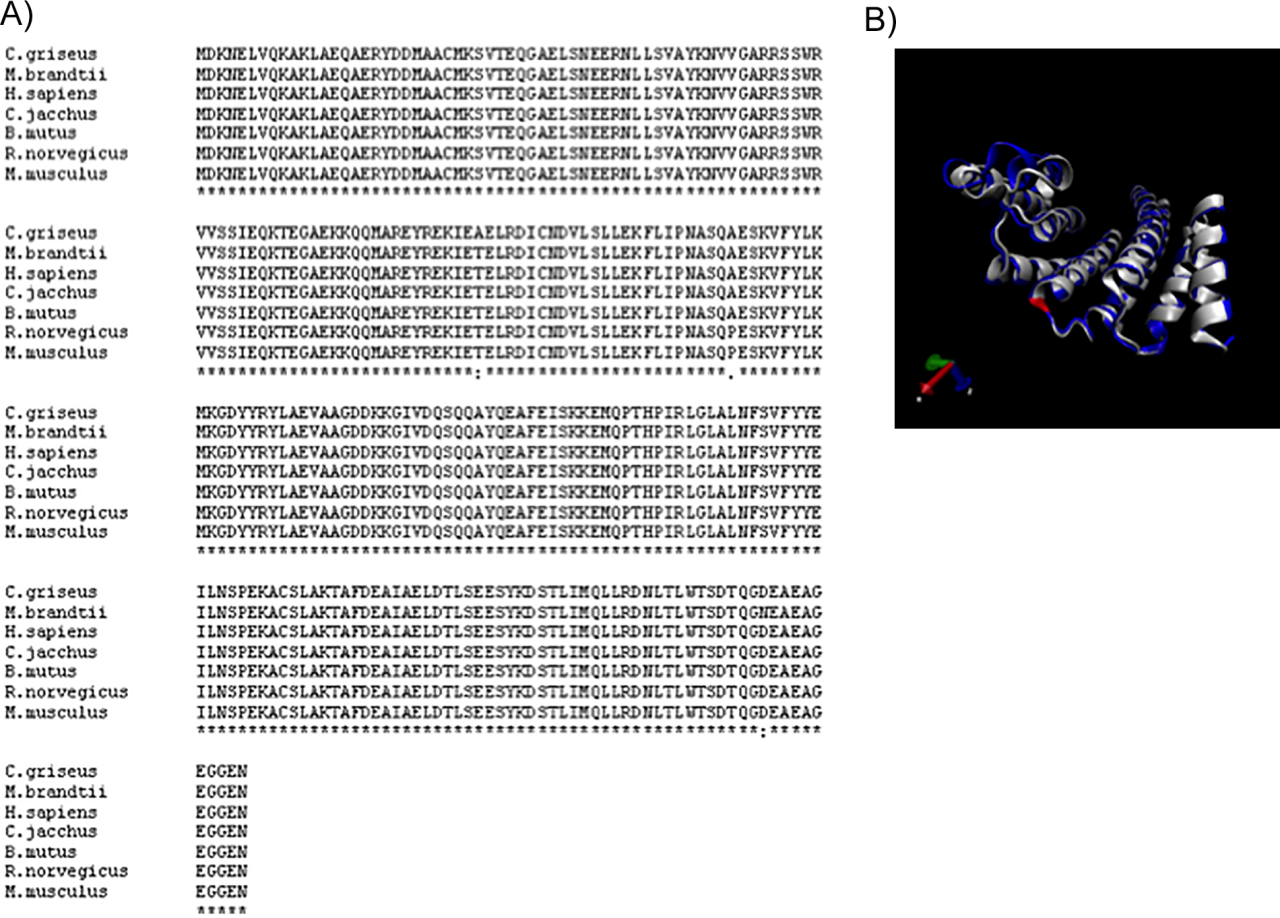
High degree of conservation for mammalian 14-3-3. (A) 14-3-3ζ protein in mammals (human, rat, mouse, bat, hamster, marmoset, and yak) show 99% conservation of sequence. Only a single amino acid difference (alanine to proline or threonine to alanine) was observed between human and rodent (mouse/rat or hamster) 14-3-3ζ. Accession numbers used for alignment are as follows—human: NP_001129171, rat: NP_037143.2, mouse: NP_035870.1, bat: XP_005879335.1, hamster: XP_003504372.1, marmoset: XP_008981647.1, yak: XP_005887072.1. (B) Structure of rat 14-3-3ζ protein **(**NP_037143.2) was simulated based on the human 14-3-3ζ structure (NP_001129171, Protein Data Bank ID-5WXN). QMEAN calculated was 0.93. Human protein (PDB ID 5D2D) is shown in blue, while rat 14-3-3ζ is shown in white. P112 of rat is shown in red color.

In comparison, each human isoform showed more conservation (>99%) when compared to its mammalian orthologs (Fig 2A). Single amino acid substitution was observed in some isoforms such as 14-3-3ζ of rat and mouse in comparison to human; however, this did not impact the structure of protein (Fig 2B). Similarly, a different single amino acid substitution was observed when human 14-3-3ζ compared with 14-3-3 of hamster.

To understand the basis of antigenicity in case of highly conserved protein sequences, we investigated the local synteny which serves as a tool to survey the extent of conservation of genetic loci during evolution[26]. All seven isoforms of 14-3-3 in human, and so in rat and mouse, albeit, segregating on different chromosomes, show significant conserved synteny in the genomic regions encompassing the gene coding for 14-3-3 across all of their genomes (Table 1, S2 Table). But synteny was not observed when chromosomal regions were compared with Xenopus or chicken. This suggested that the 14-3-3 locus diverged significantly since the evolution of mammals from amphibians and birds.

**Table 1:** Chromosomal localization of 14-3-3ζ across species.

	Chromosome	Syntenic region
**Human**	8	100,890–100,990kb
**Rat**	7	71470–71560 kb
**Mouse**	11	35905-35990kb
Drosophila*	2R	10,099-10,109kb
Chicken*	2	129,241-129,267kb

* highlight shows non-syntenic region containing 14-3-3ζ gene

### Comparisons of parasitic and human 14-3-3

**Fig 3:**
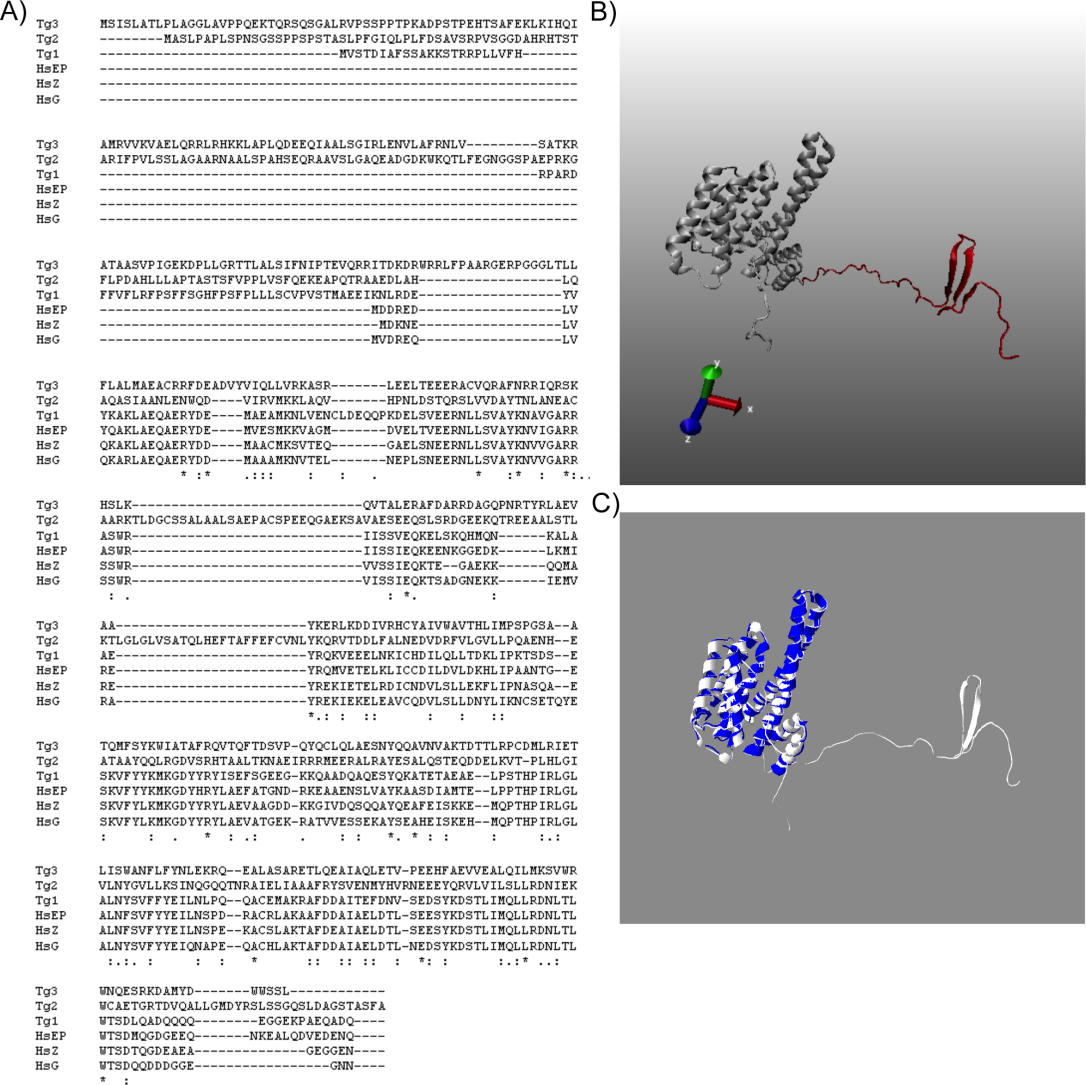
Significant difference lies between human and *T*. *gondii* 14-3-3 proteins. (A) Sequences of human 14-3-3ζ (HsZ, NP_001129171), 14-3-3ε (HsEP, NP_006752.1), and 14-3-3γ (HsG, NP_036611.2) aligned with *T*. *gondii* 14-3-3 proteins (Tg1 = XP_002365409.1, Tg2 = XP_018636600.1 Tg3 = XP_018636082.1) show areas of conservation and significant variation. An extra 62 amino acid at the N-terminal region in Tg1, and even longer N-terminal region in the case of other two family members was identified. (B) *T*. *gondii* 14-3-3 (Tg1) structure was simulated by Raptor X, using *C*. *parvum* 14-3-3 (PDB ID 2NPM) as a template. P-value of the structure is 1.62e-07. The 62aa N-terminal region is shown in red, and the rest of the protein is shown in grey. (C) Structure alignment of *T*. *gondii* 14-3-3 protein (Tg1) and human 14-3-3ε (PDB ID 2BR9). *T*. *gondii* protein is shown in white, while human protein is in blue. *T*. *gondii* structure uses the same model as in Fig 3B.

Because the antigenicity of protozoan 14-3-3 proteins is well studied [19, 27, 28], we compared protozoan 14-3-3 with its human counterpart. We identified three protein sequences using BLAST searching of the *T*. *gondii* genome (GCA_000006565.2); Tg1, Tg2 and Tg3, (TGME49_263090, TGME49_227952, TGME49_269960 respectively) that are similar to the 14-3-3 protein family. All the T. *gondii* isoforms, Tg1 (323 aa), Tg2 (476 aa) and Tg3 (422 aa) were larger compared to the human 14-3-3ε (255 aa) and human 14-3-3ζ (245 aa). All three isoforms in *T*. *gondii*, when aligned with human isoforms (ζ, ε, γ) showed an extra N-terminus sequence that varied between 62–156 amino acids, however, the C-terminus was relatively conserved (Fig 3A). The tail at the C-terminus represented an unstructured region that aligned with the variable region 6 (V6) of the 14-3-3 proteins. The extra 62 amino acid sequence on the N-terminus of Tg1 was found to be overall hydrophilic with notable presence of serine residues (9 out of 62aa). There are four putative phosphorylation sites (S10, S14, T15, T57) with the motifs KKsT and TmaE for CAMP (cAMP and cGMP-dependent protein kinase) and CK2 (Casein Kinase II) respectively. Modeling this sequence in RaptorX with a p-value = 1.62e-07, which is suggestive of highly reliable structure, showed the presence of three beta sheets (Fig 3B). Further, the homology search using *T*. *gondii* Tg1 protein (XP_002365409.1) in the non-redundant protein database (blastp 2.2.29) showed several proteins from *Neospora caninum*, *Plasmodium falciparum* and *Plutella xylostella* to be 74–77% homologous. This suggests that the overall 14-3-3 family in the protozoan has been well conserved and is different from mammalian proteins. Overlaying the structure of Tg1 with the human 14-3-3ε indicated that the most significant regions of variations between the protein structures are traceable on both the N and C-termini (Fig 3C).

In contrast to our observations with *T*. *gondii*, comparisons of the human 14-3-3 with that of parasitic worms such as *Schistosoma mansoni* showed that all six proteins of the 14-3-3 family in *S*. *mansoni* were significantly similar to human 14-3-3 isoforms. Only three isoforms (SMP 3, 4 and 6) had extra sequences at N-termini, whereas two isoforms (SMP2 and 5) were shorter than human isoforms (S2 Fig).

Further, because antibodies against 14-3-3 are observed in *Angiostrongylus cantonensis* infection of the human kidney [27], we performed blast search of the *A*. *cantonensis* genome with the nucleotide sequence of human 14-3-3. Interestingly, our analyses revealed a sequence with 80% identity to human 14-3-3ζ isoform. Both proteins showed significant homology to each other with and similarity in structural motifs (S3 Fig). A similar search conducted with the sequences of *E*. *multilocularis* also resulted in a 249 amino acid long sequence of *E*. *multilocularis* 14-3-3 (CD170865), which, when aligned with the human 14-3-3, showed maximum similarity to the epsilon isoform of 14-3-3 (NP_006752.1, data not shown).

### Comparisons of eukaryotic (yeast) 14-3-3 with human 14-3-3ε

**Fig 4:**
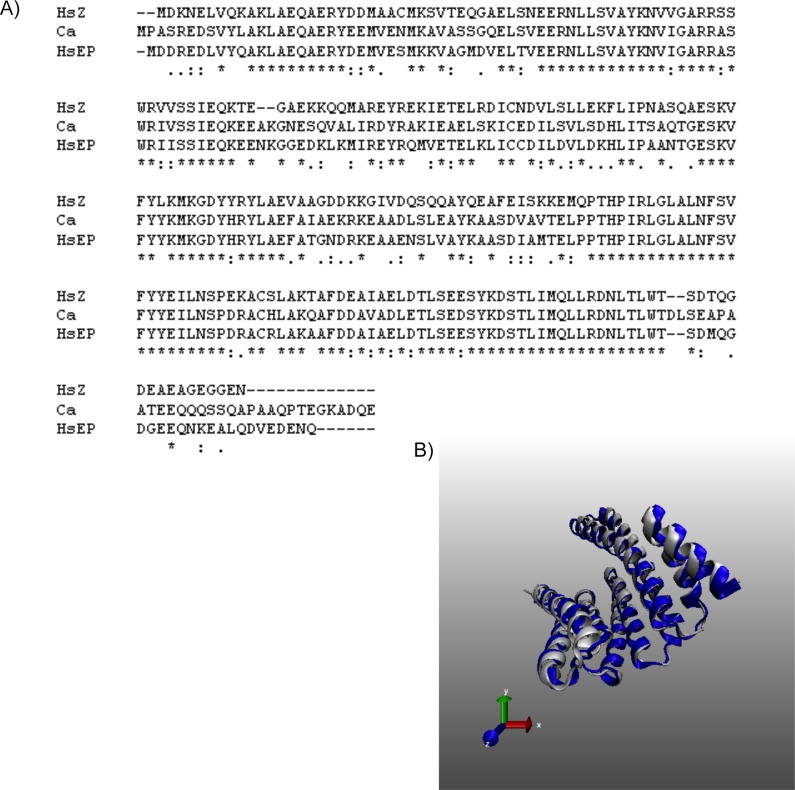
14-3-3 of *Candida albicans* and human has similar sequence and structure. Sequence alignment of *C*. *albicans* 14-3-3 (BMH) with human ζ and ε isoforms revealed 83% similarity with human ε and 79% with ζ protein. *Candida* BMH protein was simulated using *L*. *thermotolerans* 14-3-3 protein (PDB ID 5LX2) as a template. QMEAN was 0.80. *Candida* protein is shown in white, while human 14-3-3ε protein is shown in blue.

Much like in higher eukaryotes, isoforms of 14-3-3 in yeast have been shown to perform wide variety of functions[20–22]. Unlike in human and parasite, the antigenic roles of these isoforms have not been yet reported, but they are implicated in several important functions, including cell survival [29], virulence [30], regulation of gene transcription[31] and sporulation[32]. We compared the two known 14-3-3 isoforms reported in *S*. *cerevisiae* (BMH1 and BMH2), in *S*. *pombe* (Rad 24 and Rad25) and one isoform reported in *C*. *albicans (*BMH), with human 14-3-3. All the yeast homologs closely resembled human 14-3-3ε, as also reported previously [22]. BMH1 of *S*. *cerevisiae* exhibited 75% identity and 84% similarity, while BMH2 had 74% identity and 83% similarity with human 14-3-3ε. Similarly, BMH of *C*. *albicans* had 75% identity and 83% similarity with human 14-3-3ε (Fig 4A). Further, 3D structural comparison of BMH from *C*. *albicans* with human 14-3-3ε revealed an almost completely matched overlay with high QMEAN scores (QMEAN4 = 0.75 and QMEAN6 = 0.73, Fig 4B).

### Presence of 14-3-3 in bacteria

**Fig 5:**
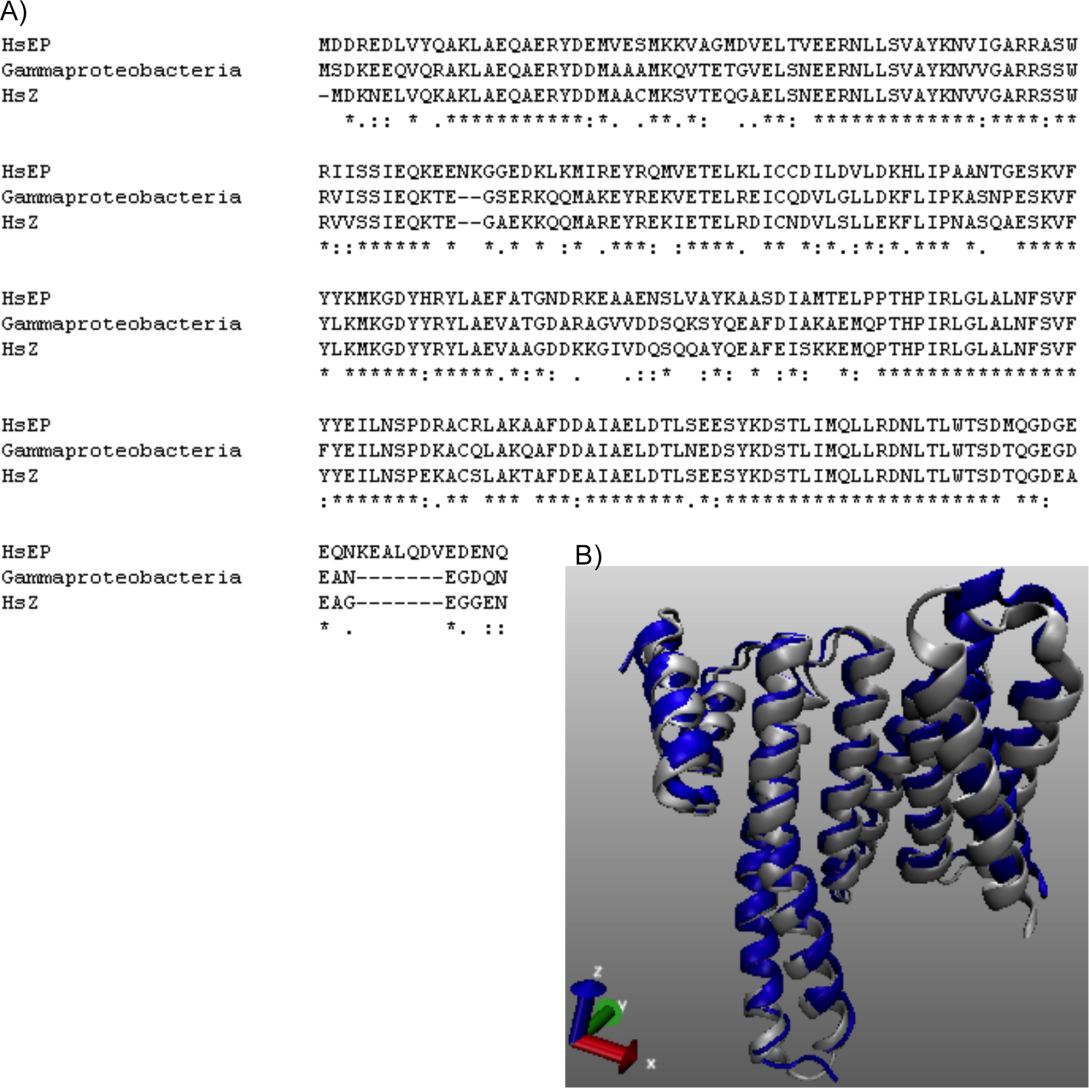
Sequence and structure of newly identified Gammaproteobacterial 14-3-3 with its human counterpart. (A) Sequence alignment of a hypothetical protein of *Gammaproteobacteria* (WP_083217732.1) with the human 14-3-3ζ and 14-3-3ε isoforms shows 90 and 82% similarity respectively. (B) Bacterial protein was simulated based upon the human 14-3-3ζ structure (NP_001129171) **(**Protein Data Bank ID 5WXN). QMEAN was 1.04. Structural alignment of a hypothetical Gammaproteobacterial 14-3-3 with the human ζ isoform (PDB ID 5D2D) shows complete overlap. Bacterial protein is shown in white color and the human protein is shown in blue.

Currently, there is no report of the presence of 14-3-3 in bacteria. However, when the sequences of human 14-3-3ε and 14-3-3ζ were blasted against bacterial databases, several hypothetical proteins with up to 50–100% similarities were identified. These proteins were identified in *Gammaproteobacteria bacterium* (2W06, WP_083217732.1), *Acinetobacter baumannii* (WP_082181584.1), *Streptomyces agglomeratus* (WP_069926675.1), and *Pseudonocardia sp*. (OJY47151.1). Furthermore, the similarity in sequence as well as structure between these bacterial proteins, in particular *Gammaproteobacteria* and human 14-3-3ζ was striking (Fig 5A and 5B). The QMEAN scores of simulated structure of bacterial 14-3-3 protein were 0.74 and 0.73 for QMEAN4 and QMEAN6 respectively. Our analyses clearly indicate the existence of 14-3-3 protein in bacteria.

### Phylogenetic analyses reveal parasitic 14-3-3 proteins as the mostly distinct

There is a significant degree of sequence conservation in the 14-3-3 family from unicellular to multicellular organisms including humans, yet only certain proteins are reported as being antigenic, is intriguing. To gain further insight into the putative antigenicity, we performed a phylogenetic analysis on the 14-3-3 proteins from phylum Chordata including mammals (*Homo sapiens* and *Mus musculus*), amphibian (*Xenopus tropicalis*), fish (*Danio rerio*), birds (*Gallus gallus*), Arthropoda (*Drosophila melanogaster*) and ascidian (*Ciona intestinalis*), plant (*Oryza sativa japonica*), several parasites from phylum including Apicomplexa (*T*. *gondii*), Platyhelminthes (*S*. *mansoni*) etc., Ascomycota (*S*. *cerevisiae*, *C*. *albicans*), and bacteria (*Gammaproteobacteria*). The phylogenetic study using PhyML program with bootstrap analysis with a value of 100, suggested that most isoforms of 14-3-3 families in mammals had an independent node. Most isoforms from human, plant, amphibian and bird tended to cluster together, but two of the plant isoforms (OsG and OsH) had a separate node. However, most parasite (*T*. *gondii*, *S*. *mansoni*), fly (*D*. *melanogaster*) and yeast (*S*. *cerevisiae*) 14-3-3 proteins maintained a separate node. In accordance to the previous report [20], our results also suggest that the epsilon isoform of 14-3-3 proteins probably separated from the rest of the family during the early phases of evolution, but remained closer to the 14-3-3 of parasites, yeast and ascidians such as *C*. *intestinalis*, and, therefore, may be considered as ancestral protein of the family. The most striking observation of this analysis was that most of the isoforms from parasites (particularly SMP6, Tg2 and Tg3) had their own independent node. The functional significance of this separation from 14-3-3 in all other organisms is yet to be investigated.

### Human 14-3-3 inhibitor is a novel antifungal agent

**Fig 6:**
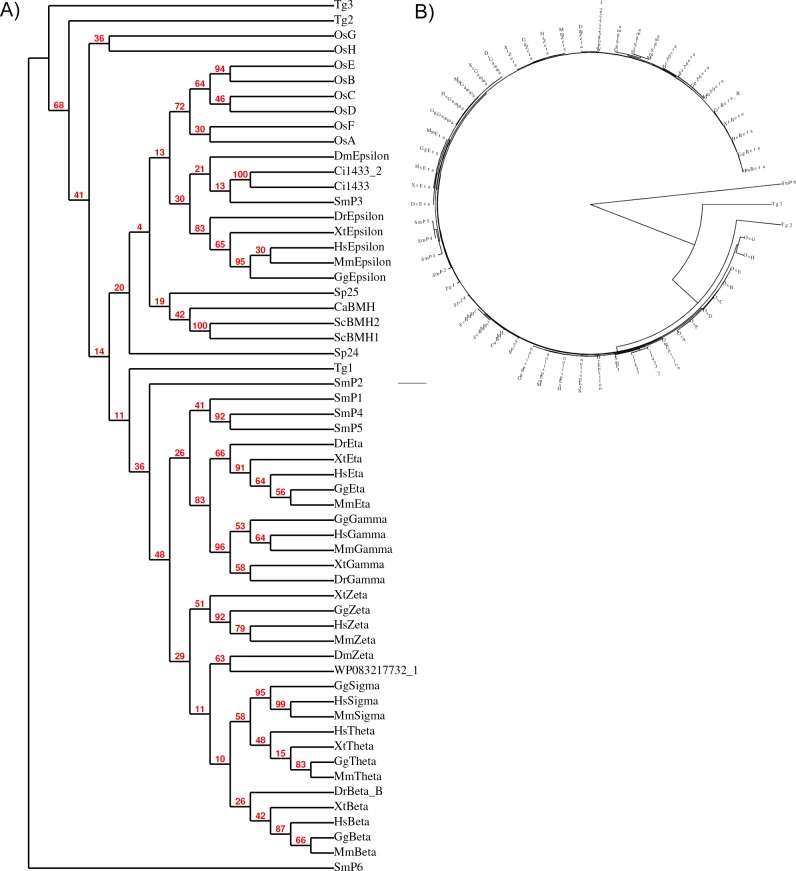
Phylogeny tree of 14-3-3 family suggests parasitic 14-3-3 diverged early on. Multiple sequence alignment from MUSCLE was used to create phylogeny tree using PhyML program with bootstrap analysis (value of 100) at www.phylogeny.fr. (A) A cladogram tree style with branch support value at each node is shown. Common node for *Candida* BMH and human epsilon proteins is shown by asterisk. (B) Circular tree emphasizes the early separation of parasitic proteins (Tg2, Tg3 and SmP6) from the rest of 14-3-3 proteins that are clustered together.

**Fig 7:**
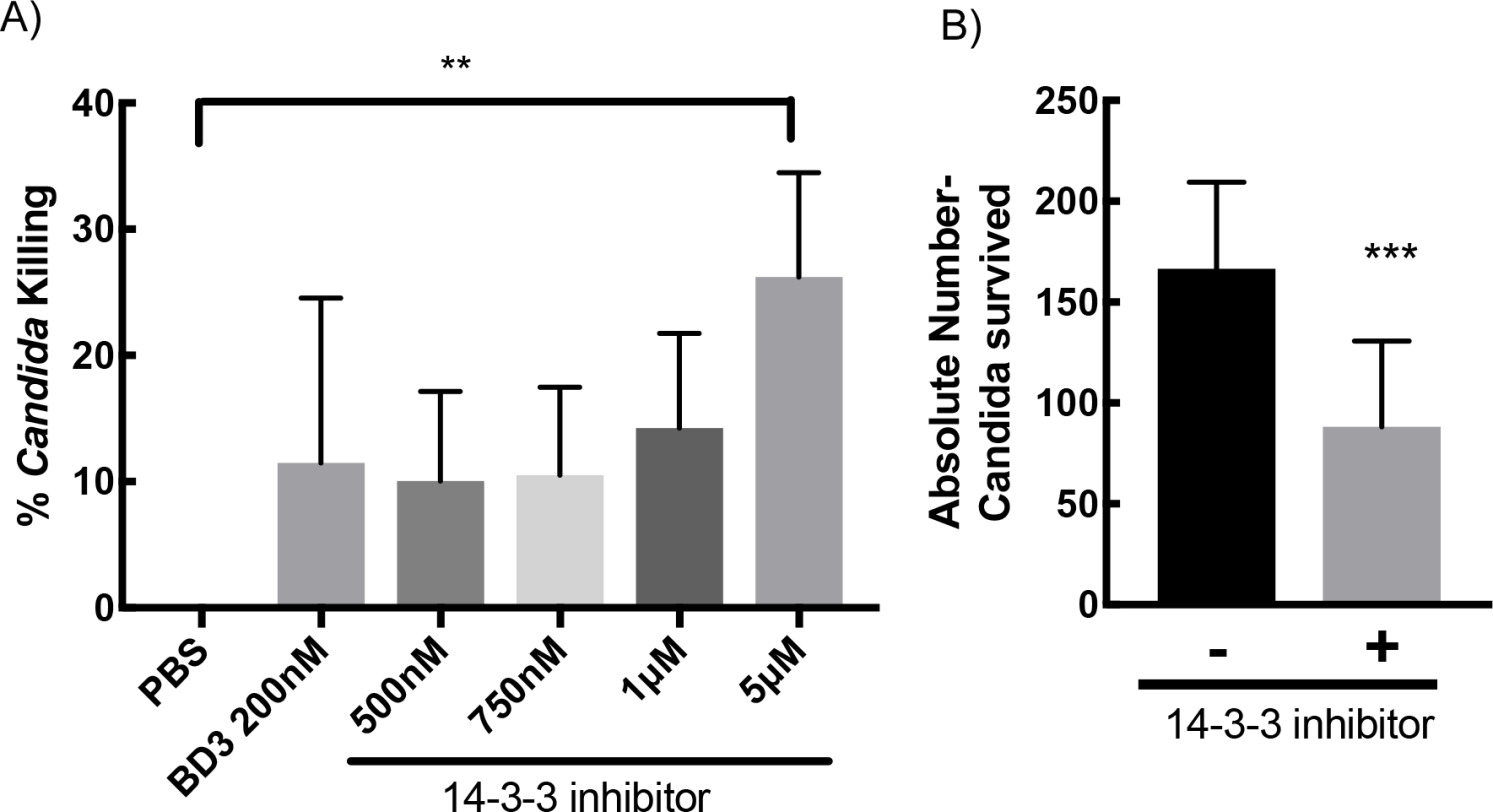
Human 14-3-3 inhibitor kills *C*. *albicans*. (A) *Candida* in exponential growth phase were subjected to 200nM of BD3 (β-defensin-3) or different doses of 14-3-3 inhibitor (R18) for 1hour. Inhibitor was then removed and cell survival was calculated as described in the method section. Significant (* p = 0.0053) cell death was observed in case of R18 treated set. (B) Incubation of TR146 cells with *Candida* for 1 hour in the presence of 1μM of BV02, another 14-3-3 inhibitor, resulted in statistically significant (*p = 0.012) reduction in colony forming units.

All of our above described analyses based on protein sequence, structure and phylogenetic status provided clues for putative, yet undiscovered, structure-function relationships of the 14-3-3 proteins. The phylogenetic analyses, wherein the positioning of the *Candida* BMH very close to human 14-3-3ε (Fig 6A and 6B), led to our hypothesis that the structural similarity between *Candida* BMH and human 14-3-3ε may indicate that a known inhibitor of human 14-3-3 would also inhibit *Candida* BMH. Because *Candida* BMH is known to be important for the survival of *C*. *albicans*, we predicted that such an inhibition would arrest the growth of *Candida*. Therefore, we tested the effect of R18, a peptide inhibitor of human 14-3-3, on the survival of *C*. *albicans*. Incubation of *C*. *albicans* with R18 resulted in a dose-dependent increase in the killing of *C*. *albicans* (Fig 7A, S4 Fig). Notably, the antifungal effect of R18 on *C*. *albicans* was as pronounced as a known inhibitor of *C*. *albicans*, β-defensin-3 (BD-3) [33, 34]. Similarly, in the infection assays using oral epithelial cells, we observed effective inhibition of *C*. *albicans* survival when BV02, another 14-3-3 inhibitor [35], was applied (Fig 7B). Furthermore, we tested the 14-3-3 inhibitor for its effect on the *S*. *cerevisiae* which also encodes two isoforms of 14-3-3 (BMH1 and BMH2) that are about 75% identical to human 14-3-3ε. These two isoforms are redundant in their function; however, the loss of BMH1 causes delays in cell cycle [36]. In phylogenetic analysis, we found all yeast 14-3-3 proteins were clustered close to each other and to human 14-3-3ε. Inspite of conserved residues that are needed for binding to R18 peptide to 14-3-3, incubation of *S*. *cerevisiae* with R18 did not result in significant killing (S4 Fig).

## Discussion

Global bioinformatic approaches to compare and contrast the similarities and differences between proteins are widely applied to investigate the antigenic nature of proteins [37]. Like many other autoantigens, the basis for the antigenicity of 14-3-3 is unknown. To explore a structure-function basis for the antigenic properties of 14-3-3 proteins, we utilized a bioinformatics based approach. In the mammalian isoforms, we identified 6 regions of variability, but these regions were highly conserved among orthologs. Phylogenetic analysis suggested that based on the sequence, the 14-3-3 family may be classified as epsilon-like and non-epsilon like, isoforms [20]. Earlier report on the 14-3-3 protein from *G*. *duodenalis*, a parasite from phylum Metamonada, also suggested its closeness to human 14-3-3ε [38]. Among the parasitic proteins, 14-3-3 from *T*. *gondii* in particular, showed unique sequences and structures, which were significantly different from that in humans. In addition to significant variation within the overlapping regions, Tg1-3 isoforms possess the additional N-terminus sequences that were strikingly different from that in mammalian 14-3-3. Our report has, for the first time, uncovered the existence of bacterial 14-3-3 in the *Gammaproteobacteria* genome, with close resemblance to human 14-3-3ζ. Also, 14-3-3 of the yeast origin showed significant similarity with the human epsilon and zeta isoforms [21, 22]. To evaluate the significance of our bioinformatic analyses, we hypothesized that structurally similar regions in two different species could be exploited for their functional interactions with known inhibitors. 14-3-3 inhibitors have been studied for promoting cell death in cancerous cells [35, 39], we noticed striking similarity between the human and yeast 14-3-3 in the key residues responsible for binding to R18 peptide, that led us to demonstrate a novel re-purposing of R18 as an antifungal agent. This was further aided by the specificity in the inhibition of *Candida* and not S. *cerevisiae*, another closely related family of yeast. Although BMH1 and BMH2 are redundant proteins that together are essential for *S*. *cerevisiae* life cycle, knockout of either of them results in delayed cell cycle [21, 22, 36]. It is unknown whether the peptide inhibitor (R18) differentially inhibits their activities. Our strategy of short-term (1 hour) inhibition of yeast 14-3-3 may also have contributed to the observed effect. Future studies will be required to specifically understand these differences.

In the case of autoimmune aortitis, the trigger of disease remains unknown. In-spite of several attempts, a microbial involvement in autoimmune aortitis remains a question [40, 41]. The presence of self-antigens that mimic bacterial pathogens has been suggested in several autoimmune diseases. This includes mycobacterial HSP in Behçet syndrome, HLA-B27 with several gram-negative bacteria (*Klebsiella pneumonia*, *Shigella* etc.) in spondyloarthropathies etc.[42]. Specific reports suggest correlated presence of autoantibody with *T*. *gondii* infection such as in giant cell arteritis (anti-14-3-3 IgG) or Wegener’s granulomatosis [19, 43]. This raised the possibility that parasitic 14-3-3 can serve as an antigen in vasculitides through molecular mimicry. Our bioinformatic studies revealed limited commonalities and significant variations between the human and *T*. *gondii* 14-3-3 proteins in both sequence and structure. This knowledge can be utilized to distinguish the antibodies against human and *T*. *gondii* 14-3-3 proteins. This leads to our speculation that protozoan (*T*. *gondii*) 14-3-3 may not be sufficient to trigger a molecular mimicry-based autoantigenic function in humans. Similarly, we observed that in-spite of lost synteny between mammals and birds, the isoforms were still clustered together in the phylogenetic analyses, thereby suggesting limited use of synteny in understanding antigenic function of 14-3-3. In contrast, the existence of a new bacterial form of 14-3-3, which is similar to the human 14-3-3, leads us to speculate that the antigenic 14-3-3 in autoimmune aortitis may be connected with bacterial infections (S5 Fig).

Along these lines, our phylogenetic analysis further showed the relative closeness between the human and *C*. *albicans* 14-3-3 [21, 22]. Although antigenic 14-3-3 of *C*. *albicans* is unknown, many human diseases with antigenic 14-3-3, such as hepatocellular carcinoma and giant cell arteritis, are associated with *Candida* infection [44–46]. This may be associated with increased susceptibility for fungal infection in the immune-compromised population [44] and infection-induced hyper activation of immune responses[46]. Based on the phylogenetic analyses of human and yeast 14-3-3, and effective inhibition of *Candida* by human 14-3-3 inhibitor, we speculate that foreign peptides (*Candida)* may invoke an autoimmune response in giant cell arteritis.

The ability of 14-3-3 proteins to homo- or hetero-dimerize regulates their functions [47], and may provide additional information related to their antigenicity. This in part is supported by Lalle et al.,[38] wherein they showed that 14-3-3 proteins from *D*. *melanogaster* and *G*. *duodenalis* are able to heterodimerize, suggesting that low level chronic infection may expose a non-self functional molecule capable of immune activation.

Beyond determining the antigenic properties of 14-3-3, the identification of specific peptide sequence similarities between the human and yeast forms of 14-3-3, presented us with a unique opportunity to contemplate the repurposing of the 14-3-3 inhibitor such as, R18, as a putative antifungal agent for *Candida*. The finding that R18 indeed can elicit a dose-dependent antifungal activity against *Candida* is not only a novel discovery by itself, but points further to evolutionarily conserved shared properties of 14-3-3 functions that may contribute to molecular mimicry type of responses in eukaryotes, resulting in autoimmunity. Thus, by the bioinformatic assessment of 14-3-3 sequences across species, our study serves as a fundamental basis towards the next steps required for identifying and understanding the new antigenic function of 14-3-3, which in turn aids to differentiate between infectious and autoimmune diseases.

## Methods

### Bioinformatic analyses

Accession numbers of genes were obtained (S1 Table) and BLAST searches were performed by the protein or nucleotide based search criteria using the basic local alignment search tool (Blast) service provided by the NCBI (https://blast.ncbi.nlm.nih.gov/Blast.cgi). Alignment was then entered into WebLogo to create the sequence logo to study conservation of sequence [25]. The Ensembl release 89 (https://www.ensembl.org/index.html) was used for mining genomic datasets. The following assemblies were used: Human (GRCh38.p10), Rat (Rnor_6.0), Mouse (GRCm38.p4), Toxoplasma (GCA_000006565.2), *Candida* (ASM18296v3), Saccharomyces (R64), Xenopus (GL172639.1), Drosophila (GCF_000001215.4) and Gammaproteobacteria (NCBI assembly- ASM171528v1, GenBank accession number-GCA_001715285.1). The website, www.Phylogeny.fr, was used to build phylogenetic tree of 14-3-3 family of proteins [48]. MUSCLE and Clustal W were used for multiple sequence alignments. SWISS-MODEL was used to simulate rat 14-3-3ζ and Gammaproteobacteria hypothetical protein structure, with PDB ID 5WXN serving as a template for both, while the crystal structure of human 14-3-3ζ was downloaded from Protein Data Bank (PDB ID 5D2D) [49]. Visualization and structure alignment was done using VMD [50]. Raptor X was used to model toxoplasma 14-3-3, with PDB ID 3M50 serving as a template [51].

### *C*. *albicans* and *S*. *cerevisiae* killing assays

Yeast cells were grown in YPD broth overnight at 30°C then subcultured to the log phase of growth [52]. The cells were washed in 10mM sodium phosphate buffer and then 10,000 cells were co-incubated with buffer, 500nM of R18 (Enzo Life Sciences, Inc.) or 200nM of BD3 (BioBasics, Inc.) as a positive control. Cells were incubated at 37°C (*C*. *albicans* CAF2-1) or 30°C (*S*. *cerevisiae* AH106) for one hour in 10mM sodium phosphate buffer, serially diluted and plated on YPD agar plates. Plates were incubated at 30°C until yeast colonies were visible for counting. Percent killing was calculated compared to a negative control containing buffer only and yeast cells.

### *Candida* killing assay with human oral epithelial cells

TR146 cells (Sigma) were grown to confluence in six well dishes using DMEM+F12 supplemented with 10% FBS and 1% pen/strep. Once confluence was reached, cells were deprived for 24 hours with DMEM supplemented with 1% BSA. After 24h, media was conditioned with 500nM of BV02. A control of unconditioned deprivation media was included as a control. After 24 hours approximately 10,000 *C*. *albicans* (CAF2-1) yeast cells that had been cultured to the log-phase of growth were added to each of the wells and incubated at 37°C for one hour. After incubation, TR146 cells and *C*. *albicans* were removed from the plate and resuspended in 10mM sodium phosphate buffer, serially diluted and plated on YPD agar plates supplemented with gentamicin and ampicillin. Plates were incubated overnight at 30°C. Plates were then counted for number of *Candida* colonies and percent killing was calculated compared to a negative control.

### Statistical analysis

For simulated structures, we used the QMEAN score (SWISS-MODEL) or probability (Raptor X), as provided by the platform utilized for determination. As suggested by Raptor X, p values less than 10^−3^ and 10^−4^ was considered significant for alpha helix and beta sheet dominant structures, respectively. For QMEAN, scores range from 0–1, with 1 being good [53]. For the survival experiments, we used two tailed unpaired t test with at least 95% confidence to determine the significance of difference.

## Supporting information

S1 Fig Sequence alignment of human isoforms. All human 14-3-3 isoforms were aligned using MUSCLE program. Fully conserved residues are marked with an asterisk (*), mostly conserved with a colon (:), weakly conserved with a period (.) and non-conserved are unlabeled. Area of variability are underlined and are numbered as v1 to v6. Labels are hsEP = ε, hsS = σ, hsG = γ, hsET = η, hsT = τ, hsZ = ζ, hsA = α.
(PDF)

S2 Fig Sequence alignment of S. mansoni vs human isoforms. Alignment of *S*. *mansoni* 14-3-3 isoforms (assembly ASM23792v2) with human 14-3-3 isoforms is shown.
(PDF)

S3 Fig Sequence alignment of Angiostrongylus vs human isoforms. Alignment of *A*. *cantonensis* 14-3-3 with human zeta isoform showed significant homology between the two proteins.
(PDF)

S4 Fig Effect of 14-3-3 inhibitor on the yeast growth. (A) Inhibition of 14-3-3 in *S*. *cerevisiae* does not decrease cell survivality. Unlike *C*. *albicans*, Inhibition of 14-3-3 by peptide inhibitor R18 (500nM) did not result in statistically significant change in the yeast survival in comparison to b-defensin 3 (BD3, 200nM). (B) Representative Images from the *Candida* survival experiment (Fig 7A) are shown. Number of counted colonies on each plate are listed below.
(PDF)

S5 Fig Alignment of all 14-3-3 genes investigated in the current study. A table showing abbreviations that are used in alignment file, followed by alignment file.
(PDF)

S1 Table List of 14-3-3 proteins studied. Accession Numbers of All 14-3-3 Proteins. obtained from NCBI, are listed.
(PDF)

S2 Table Chromosomal location of 14-3-3. Position of 14-3-3 isoforms In different species is listed.
(PDF)

## References

[1] Morrison DK. The 14-3-3 proteins: integrators of diverse signaling cues that impact cell fate and cancer development. Trends Cell Biol. 2009;19(1):16–23. Epub 2008/11/26. doi: 10.1016/j.tcb.2008.10.003 ; PubMed Central PMCID: PMC3073487. 19027299 10.1016/j.tcb.2008.10.003 PMC3073487

[2] Tsai HC, Huang YL, Chen YS, Yen CM, Tsai R, Lee SS, et al 14-3-3beta protein expression in eosinophilic meningitis caused by Angiostrongylus cantonensis infection. BMC Res Notes. 2014;7:97 doi: 10.1186/1756-0500-7-97 ; PubMed Central PMCID: PMCPMC3932789. 24555778 10.1186/1756-0500-7-97 PMC3932789

[3] Zhao J, Meyerkord CL, Du Y, Khuri FR, Fu H. 14-3-3 proteins as potential therapeutic targets. Semin Cell Dev Biol. 2011;22(7):705–12. Epub 2011/10/11. doi: 10.1016/j.semcdb.2011.09.012 ; PubMed Central PMCID: PMC3207012. 21983031 10.1016/j.semcdb.2011.09.012 PMC3207012

[4] Kilani RT, Maksymowych WP, Aitken A, Boire G, St-Pierre Y, Li Y, et al Detection of high levels of 2 specific isoforms of 14-3-3 proteins in synovial fluid from patients with joint inflammation. The Journal of rheumatology. 2007;34(8):1650–7. Epub 2007/07/06. . 17611984

[5] Hatzipetros I, Gocze P, Koszegi T, Jaray A, Szereday L, Polgar B, et al Investigating the clinical potential for 14-3-3 zeta protein to serve as a biomarker for epithelial ovarian cancer. J Ovarian Res. 2013;6(1):79 Epub 2013/11/19. doi: 10.1186/1757-2215-6-79 ; PubMed Central PMCID: PMC3835546. 24238270 10.1186/1757-2215-6-79 PMC3835546

[6] Foote M, Zhou Y. 14-3-3 proteins in neurological disorders. Int J Biochem Mol Biol. 2012;3(2):152–64. Epub 2012/07/10. ; PubMed Central PMCID: PMC3388734. 22773956 PMC3388734

[7] Maksymowych WP, Marotta A. 14-3-3eta: a novel biomarker platform for rheumatoid arthritis. Clinical and experimental rheumatology. 2014;32(5 Suppl 85):S-35-9. . 25365087

[8] Colucci M, Roccatagliata L, Capello E, Narciso E, Latronico N, Tabaton M, et al The 14-3-3 protein in multiple sclerosis: a marker of disease severity. Mult Scler. 2004;10(5):477–81. doi: 10.1191/1352458504ms1089oa . 15471360 10.1191/1352458504ms1089oa

[9] Shandala T, Woodcock JM, Ng Y, Biggs L, Skoulakis EM, Brooks DA, et al Drosophila 14-3-3epsilon has a crucial role in anti-microbial peptide secretion and innate immunity. J Cell Sci. 2011;124(Pt 13):2165–74. Epub 2011/06/15. doi: 10.1242/jcs.080598 . 21670199 10.1242/jcs.080598

[10] Liu HM, Loo YM, Horner SM, Zornetzer GA, Katze MG, Gale M Jr., The mitochondrial targeting chaperone 14-3-3epsilon regulates a RIG-I translocon that mediates membrane association and innate antiviral immunity. Cell Host Microbe. 2012;11(5):528–37. Epub 2012/05/23. doi: 10.1016/j.chom.2012.04.006 ; PubMed Central PMCID: PMC3358705. 22607805 10.1016/j.chom.2012.04.006 PMC3358705

[11] Ulvila J, Vanha-aho LM, Kleino A, Vaha-Makila M, Vuoksio M, Eskelinen S, et al Cofilin regulator 14-3-3zeta is an evolutionarily conserved protein required for phagocytosis and microbial resistance. J Leukoc Biol. 2011;89(5):649–59. Epub 2011/01/07. doi: 10.1189/jlb.0410195 . 21208897 10.1189/jlb.0410195

[12] Lozano-Duran R, Robatzek S. 14-3-3 proteins in plant-pathogen interactions. Mol Plant Microbe Interact. 2015;28(5):511–8. doi: 10.1094/MPMI-10-14-0322-CR . 25584723 10.1094/MPMI-10-14-0322-CR

[13] Chakravarti R, Gupta K, Swain M, Willard B, Scholtz J, Svensson LG, et al 14-3-3 in Thoracic Aortic Aneurysms: Identification of a Novel Autoantigen in Large Vessel Vasculitis. Arthritis & rheumatology (Hoboken, NJ). 2015;67(7):1913–21. Epub 2015/04/29. doi: 10.1002/art.39130 ; PubMed Central PMCID: PMCPmc4624269. 25917817 10.1002/art.39130 PMC4624269

[14] Neal CL, Yu D. 14-3-3zeta as a prognostic marker and therapeutic target for cancer. Expert Opin Ther Targets. 2010;14(12):1343–54. doi: 10.1517/14728222.2010.531011 ; PubMed Central PMCID: PMCPMC3017465. 21058923 10.1517/14728222.2010.531011 PMC3017465

[15] Maksymowych WP, Boire G, van Schaardenburg D, Wichuk S, Turk S, Boers M, et al 14-3-3eta Autoantibodies: Diagnostic Use in Early Rheumatoid Arthritis. The Journal of rheumatology. 2015;42(9):1587–94. doi: 10.3899/jrheum.141385 . 26178283 10.3899/jrheum.141385

[16] Siles-Lucas M, Merli M, Gottstein B. 14-3-3 proteins in Echinococcus: their role and potential as protective antigens. Exp Parasitol. 2008;119(4):516–23. doi: 10.1016/j.exppara.2008.01.009 . 18316081 10.1016/j.exppara.2008.01.009

[17] Assossou O, Besson F, Rouault JP, Persat F, Ferrandiz J, Mayencon M, et al Characterization of an excreted/secreted antigen form of 14-3-3 protein in Toxoplasma gondii tachyzoites. FEMS Microbiol Lett. 2004;234(1):19–25. doi: 10.1016/j.femsle.2004.02.024 . 15109715 10.1016/j.femsle.2004.02.024

[18] Liu M, Liu X, Ren P, Li J, Chai Y, Zheng SJ, et al A cancer-related protein 14-3-3zeta is a potential tumor-associated antigen in immunodiagnosis of hepatocellular carcinoma. Tumour Biol. 2014;35(5):4247–56. doi: 10.1007/s13277-013-1555-8 ; PubMed Central PMCID: PMCPMC4096569. 24390614 10.1007/s13277-013-1555-8 PMC4096569

[19] Kistner A, Bigler MB, Glatz K, Egli SB, Baldin FS, Marquardsen FA, et al Characteristics of autoantibodies targeting 14-3-3 proteins and their association with clinical features in newly diagnosed giant cell arteritis. Rheumatology (Oxford, England). 2017 Epub 2017/01/09. doi: 10.1093/rheumatology/kew469 . 28064210 10.1093/rheumatology/kew469

[20] Wang W, Shakes DC. Molecular evolution of the 14-3-3 protein family. J Mol Evol. 1996;43(4):384–98. . 8798343 10.1007/BF02339012

[21] van Heusden GP, Steensma HY. Yeast 14-3-3 proteins. Yeast. 2006;23(3):159–71. doi: 10.1002/yea.1338 . 16498703 10.1002/yea.1338

[22] Kumar R. An account of fungal 14-3-3 proteins. Eur J Cell Biol. 2017;96(2):206–17. doi: 10.1016/j.ejcb.2017.02.006 . 28258766 10.1016/j.ejcb.2017.02.006

[23] Tzivion G, Shen YH, Zhu J. 14-3-3 proteins; bringing new definitions to scaffolding. Oncogene. 2001;20(44):6331–8. doi: 10.1038/sj.onc.1204777 . 11607836 10.1038/sj.onc.1204777

[24] Rosenquist M, Sehnke P, Ferl RJ, Sommarin M, Larsson C. Evolution of the 14-3-3 protein family: does the large number of isoforms in multicellular organisms reflect functional specificity? J Mol Evol. 2000;51(5):446–58. . 11080367 10.1007/s002390010107

[25] Crooks GE, Hon G, Chandonia JM, Brenner SE. WebLogo: a sequence logo generator. Genome Res. 2004;14(6):1188–90. doi: 10.1101/gr.849004 ; PubMed Central PMCID: PMCPMC419797. 15173120 10.1101/gr.849004 PMC419797

[26] Chakravarti R, Adams JC. Comparative genomics of the syndecans defines an ancestral genomic context associated with matrilins in vertebrates. BMC Genomics. 2006;7:83 doi: 10.1186/1471-2164-7-83 ; PubMed Central PMCID: PMCPMC1464127. 16620374 10.1186/1471-2164-7-83 PMC1464127

[27] Morassutti AL, Levert K, Perelygin A, da Silva AJ, Wilkins P, Graeff-Teixeira C. The 31-kDa antigen of Angiostrongylus cantonensis comprises distinct antigenic glycoproteins. Vector Borne Zoonotic Dis. 2012;12(11):961–8. doi: 10.1089/vbz.2011.0957 ; PubMed Central PMCID: PMCPMC3491624. 22925026 10.1089/vbz.2011.0957 PMC3491624

[28] Duarte J, Herbert F, Guiyedi V, Franetich JF, Roland J, Cazenave PA, et al High levels of immunoglobulin E autoantibody to 14-3-3 epsilon protein correlate with protection against severe Plasmodium falciparum malaria. J Infect Dis. 2012;206(11):1781–9. doi: 10.1093/infdis/jis595 . 22984113 10.1093/infdis/jis595

[29] Cognetti D, Davis D, Sturtevant J. The Candida albicans 14-3-3 gene, BMH1, is essential for growth. Yeast. 2002;19(1):55–67. doi: 10.1002/yea.804 . 11754483 10.1002/yea.804

[30] Kelly MN, Johnston DA, Peel BA, Morgan TW, Palmer GE, Sturtevant JE. Bmh1p (14-3-3) mediates pathways associated with virulence in Candida albicans. Microbiology. 2009;155(Pt 5):1536–46. doi: 10.1099/mic.0.027532-0 ; PubMed Central PMCID: PMCPMC2772093. 19372164 10.1099/mic.0.027532-0 PMC2772093

[31] Parua PK, Ratnakumar S, Braun KA, Dombek KM, Arms E, Ryan PM, et al 14-3-3 (Bmh) proteins inhibit transcription activation by Adr1 through direct binding to its regulatory domain. Mol Cell Biol. 2010;30(22):5273–83. doi: 10.1128/MCB.00715-10 ; PubMed Central PMCID: PMCPMC2976377. 20855531 10.1128/MCB.00715-10 PMC2976377

[32] Slubowski CJ, Paulissen SM, Huang LS. The GCKIII kinase Sps1 and the 14-3-3 isoforms, Bmh1 and Bmh2, cooperate to ensure proper sporulation in Saccharomyces cerevisiae. PLoS One. 2014;9(11):e113528 doi: 10.1371/journal.pone.0113528 ; PubMed Central PMCID: PMCPMC4237420. 25409301 10.1371/journal.pone.0113528 PMC4237420

[33] Feng Z, Jiang B, Chandra J, Ghannoum M, Nelson S, Weinberg A. Human beta-defensins: differential activity against candidal species and regulation by Candida albicans. J Dent Res. 2005;84(5):445–50. doi: 10.1177/154405910508400509 . 15840781 10.1177/154405910508400509

[34] Conti HR, Shen F, Nayyar N, Stocum E, Sun JN, Lindemann MJ, et al Th17 cells and IL-17 receptor signaling are essential for mucosal host defense against oral candidiasis. J Exp Med. 2009;206(2):299–311. doi: 10.1084/jem.20081463 ; PubMed Central PMCID: PMCPMC2646568. 19204111 10.1084/jem.20081463 PMC2646568

[35] Mancini M, Corradi V, Petta S, Barbieri E, Manetti F, Botta M, et al A new nonpeptidic inhibitor of 14-3-3 induces apoptotic cell death in chronic myeloid leukemia sensitive or resistant to imatinib. J Pharmacol Exp Ther. 2011;336(3):596–604. doi: 10.1124/jpet.110.172536 . 21041536 10.1124/jpet.110.172536

[36] Lottersberger F, Rubert F, Baldo V, Lucchini G, Longhese MP. Functions of Saccharomyces cerevisiae 14-3-3 proteins in response to DNA damage and to DNA replication stress. Genetics. 2003;165(4):1717–32. ; PubMed Central PMCID: PMCPMC1462906. 14704161 10.1093/genetics/165.4.1717 PMC1462906

[37] Lu G, Villalba M, Coscia MR, Hoffman DR, King TP. Sequence analysis and antigenic cross-reactivity of a venom allergen, antigen 5, from hornets, wasps, and yellow jackets. J Immunol. 1993;150(7):2823–30. . 8454859

[38] Lalle M, Leptourgidou F, Camerini S, Pozio E, Skoulakis EM. Interkingdom complementation reveals structural conservation and functional divergence of 14-3-3 proteins. PLoS One. 2013;8(10):e78090 doi: 10.1371/journal.pone.0078090 ; PubMed Central PMCID: PMCPMC3795638. 24147113 10.1371/journal.pone.0078090 PMC3795638

[39] Masters SC, Fu H. 14-3-3 proteins mediate an essential anti-apoptotic signal. J Biol Chem. 2001;276(48):45193–200. doi: 10.1074/jbc.M105971200 . 11577088 10.1074/jbc.M105971200

[40] Gilden D, White T, Khmeleva N, Katz BJ, Nagel MA. Blinded search for varicella zoster virus in giant cell arteritis (GCA)-positive and GCA-negative temporal arteries. J Neurol Sci. 2016;364:141–3. doi: 10.1016/j.jns.2016.03.020 ; PubMed Central PMCID: PMCPMC4834150. 27084233 10.1016/j.jns.2016.03.020 PMC4834150

[41] Sammel AM, Rosettenstein K, Post JJ, Bertouch JV. Limited evidence of active varicella zoster virus (VZV) infection in a cohort of acute giant cell arteritis (GCA) patients. Annals of the Rheumatic Diseases. 2016;75:567.

[42] Cusick MF, Libbey JE, Fujinami RS. Molecular mimicry as a mechanism of autoimmune disease. Clin Rev Allergy Immunol. 2012;42(1):102–11. doi: 10.1007/s12016-011-8293-8 ; PubMed Central PMCID: PMCPMC3266166. 22095454 10.1007/s12016-011-8294-7 PMC3266166

[43] Lidar M, Lipschitz N, Langevitz P, Barzilai O, Ram M, Porat-Katz BS, et al. Infectious serologies and autoantibodies in wegener's granulomatosis and other vasculitides: Novel associations disclosed using the rad BioPlex 2200. 2009. p. 649–57. 10.1111/j.1749-6632.2009.04641.x 19758211

[44] Mohd Bakri M, Mohd Hussaini H, Rachel Holmes A, David Cannon R, Mary Rich A. Revisiting the association between candidal infection and carcinoma, particularly oral squamous cell carcinoma. J Oral Microbiol. 2010;2 doi: 10.3402/jom.v2i0.5780 ; PubMed Central PMCID: PMCPMC3084579. 21523221 10.3402/jom.v2i0.5780 PMC3084579

[45] Grignetti M, Carraro M, Faccini L. [An atypical presentation of a case of Horton's giant-cell arteritis]. Minerva Med. 1995;86(12):551–3. . 8684682

[46] Kumamoto CA. Inflammation and gastrointestinal Candida colonization. Curr Opin Microbiol. 2011;14(4):386–91. doi: 10.1016/j.mib.2011.07.015 ; PubMed Central PMCID: PMCPMC3163673. 21802979 10.1016/j.mib.2011.07.015 PMC3163673

[47] Yang X, Lee WH, Sobott F, Papagrigoriou E, Robinson CV, Grossmann JG, et al Structural basis for protein-protein interactions in the 14-3-3 protein family. Proc Natl Acad Sci U S A. 2006;103(46):17237–42. doi: 10.1073/pnas.0605779103 ; PubMed Central PMCID: PMCPMC1859916. 17085597 10.1073/pnas.0605779103 PMC1859916

[48] Dereeper A, Guignon V, Blanc G, Audic S, Buffet S, Chevenet F, et al Phylogeny.fr: robust phylogenetic analysis for the non-specialist. Nucleic Acids Res. 2008;36(Web Server issue):W465–9. doi: 10.1093/nar/gkn180 ; PubMed Central PMCID: PMCPMC2447785. 18424797 10.1093/nar/gkn180 PMC2447785

[49] Guex N, Peitsch MC, Schwede T. Automated comparative protein structure modeling with SWISS-MODEL and Swiss-PdbViewer: a historical perspective. Electrophoresis. 2009;30 Suppl 1:S162–73. doi: 10.1002/elps.200900140 . 19517507 10.1002/elps.200900140

[50] Humphrey W, Dalke A, Schulten K. VMD: visual molecular dynamics. J Mol Graph. 1996;14(1):33–8, 27–8. . 8744570 10.1016/0263-7855(96)00018-5

[51] Kallberg M, Wang H, Wang S, Peng J, Wang Z, Lu H, et al Template-based protein structure modeling using the RaptorX web server. Nat Protoc. 2012;7(8):1511–22. doi: 10.1038/nprot.2012.085 ; PubMed Central PMCID: PMCPMC4730388. 22814390 10.1038/nprot.2012.085 PMC4730388

[52] Conti HR, Baker O, Freeman AF, Jang WS, Holland SM, Li RA, et al New mechanism of oral immunity to mucosal candidiasis in hyper-IgE syndrome. Mucosal Immunol. 2011;4(4):448–55. doi: 10.1038/mi.2011.5 ; PubMed Central PMCID: PMCPMC3119375. 21346738 10.1038/mi.2011.5 PMC3119375

[53] Benkert P, Tosatto SC, Schomburg D. QMEAN: A comprehensive scoring function for model quality assessment. Proteins. 2008;71(1):261–77. doi: 10.1002/prot.21715 . 17932912 10.1002/prot.21715

