# Supplementary material for: Bioinformatic analysis reveals new determinants of antigenic 14-3-3 proteins and a novel antifungal strategy
Source: PLoS One. 2017 Dec 12;12(12):e0189503. doi: 10.1371/journal.pone.0189503 (PMC5726717; doi:10.1371/journal.pone.0189503)
Supplement: S1 Fig — All human 14-3-3 isoforms were aligned using MUSCLE program. Fully conserved residues are marked with an asterisk (*), mostly conserved with a colon (:), weakly conserved with a period (.) and non-conserved are unlabeled. Area of variability are underlined and are numbered as v1 to v6. Labels are hsEP = ε, hsS = σ, hsG = γ, hsET = η, hsT = τ, hsZ = ζ, hsA = α. (PDF) [file pone.0189503.s001.pdf]

Supplemental Fig 1: Sequence Alignment of Human Isoforms. Variable regions (v1-v6) are underlined.

```
HsEP    -MDDREDLVYQAKLAEQAERYDEMVESMKKVAGMDVELTVEERNLLSVAYKNVIGARRAS
HsS     --MERASLIQKAKLAEQAERYEDMAAFMKGAVEKGEELSCEERNLLSVAYKNVVGGQRAA
HsG     -MVDREQLVQKARLAEQAERYDDMAAAMKNVTELNEPLSNEERNLLSVAYKNVVGARRSS
HsET    -MGDREQLLQRARLAEQAERYDDMASAMKAVTELNEPLSNEDRNLLSVAYKNVVGARRSS
HsT     --MEKTELIQKAKLAEQAERYDDMATCMKAVTEQGAELSNEERNLLSVAYKNVVGGRRSA
HsZ     --MDKNELVQKAKLAEQAERYDDMAACMKSVTEQGAELSNEERNLLSVAYKNVVGARRSS
HsA     MTMDKSELVQKAKLAEQAERYDDMAAAMKAVTEQGHELSNEERNLLSVAYKNVVGARRSS
           :. .*: .*.********::*.  ** ..  .  *: *:***********:*..*::
           V1                        V2
HsEP    WRIISSIEQKEENKGGEDKLKMIREYRQMVETELKLICCDILDVLDKHLIPAANTG--ES
HsS     WRVLSSIEQKSNEEGSEEKGPEVREYREKVETELQGVCDTVLGLLDSHLIKEAGDA--ES
HsG     WRVISSIEQKTSADGNEKKIEMVRAYREKIEKELEAVCQDVLSLLDNYLIKNCSETQYES
HsET    WRVISSIEQKTMADGNEKKLEKVKAYREKIEKELETVCNDVLSLLDKFLIKNCNDFQYES
HsT     WRVISSIEQKT--DTSDKKLQLIKDYREKVESELRSICTTVLELLDKYLIANATNP--ES
HsZ     WRVVSSIEQKT--EGAEKKQQMAREYREKIETELRDICNDVLSLLEKFLIPNASQA--ES
HsA     WRVISSIEQKT--ERNEKKQQMGKEYREKIEAELQDICNDVLELLDKYLIPNATQP--ES
        **::******   .  :.*    . **: :* **  :*  :* :*:..** .      **
                         V3                          V4
HsEP    KVFYYKMKGDYHRYLAEFATGNDRKEAAENSLVAYKAASDIAMTELPPTHPIRLGLALNF
HsS     RVFYLKMKGDYYRYLAEVATGDDKKRIIDSARSAYQEAMDISKKEMPPTNPIRLGLALNF
HsG     KVFYLKMKGDYYRYLAEVATGEKRATVVESSEKAYSEAHEISKEHMQPTHPIRLGLALNY
HsET    KVFYLKMKGDYYRYLAEVASGEKKNSVVEASEAAYKEAFEISKEQMQPTHPIRLGLALNF
HsT     KVFYLKMKGDYFRYLAEVACGDDRKQTIDNSQGAYQEAFDISKKEMQPTHPIRLGLALNF
HsZ     KVFYLKMKGDYYRYLAEVAAGDDKKGIVDQSQQAYQEAFEISKKEMQPTHPIRLGLALNF
HsA     KVFYLKMKGDYFRYLSEVASGDNKQTTVSNSQQAYQEAFEISKKEMQPTHPIRLGLALNF
        .*** ******.***:*.* *:..    . :  **. * :*:   : **:*********:
                                V5
HsEP    SVFYYEILNSPDRACRLAKAAFDDAIAELDTLSEESYKDSTLIMQLLRDNLTLWTSDMQG
HsS     SVFHYEIANSPEEAISLAKTTFDEAMADLHTLSEDSYKDSTLIMQLLRDNLTLWTADNAG
HsG     SVFYYEIQNAPEQACHLAKTAFDDAIAELDTLNEDSYKDSTLIMQLLRDNLTLWTSDQQD
HsET    SVFYYEIQNAPEQACLLAKQAFDDAIAELDTLNEDSYKDSTLIMQLLRDNLTLWTSDQQD
HsT     SVFYYEILNNPELACTLAKTAFDEAIAELDTLNEDSYKDSTLIMQLLRDNLTLWTSDSAG
HsZ     SVFYYEILNSPEKACSLAKTAFDEAIAELDTLSEESYKDSTLIMQLLRDNLTLWTSDTQG
HsA     SVFYYEILNSPEKACSLAKTAFDEAIAELDTLNEESYKDSTLIMQLLRDNLTLWTSENQG
        ***:*** * *: *  *** :**:*:*:* **.*:*********************::  .

HsEP    DGEEQNKEALQDVEDENQ
HsS     EEG--GEAP----QEPQS
HsG     DDG--GEGN-----N---
HsET    EEA--GEGN---------
HsT     EECDAAEGA----EN---
HsZ     DEAEAGEGG----EN---
HsA     DEGDAGEGE-----N---
        :     :
               V6
```
