# Supplementary material for: Bioinformatic analysis reveals new determinants of antigenic 14-3-3 proteins and a novel antifungal strategy
Source: PLoS One. 2017 Dec 12;12(12):e0189503. doi: 10.1371/journal.pone.0189503 (PMC5726717; doi:10.1371/journal.pone.0189503)
Supplement: S2 Fig — Alignment of S. mansoni 14-3-3 isoforms (assembly ASM23792v2) with human 14-3-3 isoforms is shown. (PDF) [file pone.0189503.s002.pdf]

```
SMP5            ----------------------MLC-----------------IYTLLPCNNSPEALASHH
SMP3            MCDDSWLNESSIKDKDSLITLAKIQEQAERFNDMACAMKKV-VETSKTLNNEERNLFSVA
Smp2            ---------------------------------MVDAMKEV-VEMAEELTVEERNLLSVA
Smp1            ----------MDELRERNIVLAKLCEQAERYDEMVKAMIEIATNTETELTVEERNLLSVA
SMP4            -MTTSWVLQSKDLSNTDLVHIAKLAEQAERYDDMAAAMKRY-TEASGNLGNEERNLLSVA
SMP6            -MTTSWVTQCEDLSNTDLVHIAKLAEQAERYDDMAAAMKRY-TEASGTLGNEERNLLSVA
hsEP            -----------MDDREDLVYQAKLAEQAERYDEMVESMKKV-AGMDVELTVEERNLLSVA
hsS             ------------MERASLIQKAKLAEQAERYEDMAAFMKGA-VEKGEELSCEERNLLSVA
hsG             -----------MVDREQLVQKARLAEQAERYDDMAAAMKNV-TELNEPLSNEERNLLSVA
hsET            -----------MGDREQLLQRARLAEQAERYDDMASAMKAV-TELNEPLSNEDRNLLSVA
hsT             ------------MEKTELIQKAKLAEQAERYDDMATCMKAV-TEQGAELSNEERNLLSVA
hsZ             ------------MDKNELVQKAKLAEQAERYDDMAACMKSV-TEQGAELSNEERNLLSVA
hsA             ----------MTMDKSELVQKAKLAEQAERYDDMAAAMKAV-TEQGHELSNEERNLLSVA
                                                                   .   * *

SMP5            LRYLIYSPESILHILPIQLNGVVVLITDQAIWCIRD-----------------NSLQNWI
SMP3            YKNVVGCCRSAWRVVSNIEQRL----DDQKKKQAGEYRSTIEKELQAVCQEVLDLLHESL
Smp2            YKNVIGSRRSSWRVFSAVEQTEGNRGNAEKQACAKKFREVLESELDRVSKDILELIDKYL
Smp1            YKNVIGARRSSWRIINSKESQDEAKGSD-EIHITKRFRKEVEKELDEICTSILNLLDNCL
SMP4            YKNVVGARRSAWRVIHGSEMKAVNDRT--KKQIAEEYRIKMEKELNTICNQVLALLEDYL
SMP6            YKNVVGARRSAWRVISGSETKAANDHM--KSQIAEEYRIKIEKELNAICDQVLVLLKDYL
hsEP            YKNVIGARRASWRIISSIEQKEENKGGEDKLKMIREYRQMVETELKLICCDILDVLDKHL
hsS             YKNVVGGQRAAWRVLSSIEQKSNEEGSEEKGPEVREYREKVETELQGVCDTVLGLLDSHL
hsG             YKNVVGARRSSWRVISSIEQKTSADGNEKKIEMVRAYREKIEKELEAVCQDVLSLLDNYL
hsET            YKNVVGARRSSWRVISSIEQKTMADGNEKKLEKVKAYREKIEKELETVCNDVLSLLDKFL
hsT             YKNVVGGRRSAWRVISSIEQKT--DTSDKKLQLIKDYREKVESELRSICTTVLELLDKYL
hsZ             YKNVVGARRSSWRVVSSIEQKT--EGAEKKQQMAREYREKIETELRDICNDVLSLLEKFL
hsA             YKNVVGARRSSWRVISSIEQKT--ERNEKKQQMGKEYREKIEAELQDICNDVLELLDKYL
                 . ::    :  .:.                                        : . :

SMP5            TSRNPVGNNNTKLSFLAWMNNSHGFHKQNNNGNN----------------SDGNEKLRGM
SMP3            LKSENTA--EGFVFYKKMEGDYYRYLAEVLTGDKSADVVKHSREAYQAATEKANSDLPPT
Smp2            IKSATKS--DSKVFYLKMKGDYFRYMAEFSVDPQRKKAAEESNKAYQEASEIAATQLFPT
Smp1            LPKAVSD--ESKVFLNKMRGDYHRYRAEYSVGNQRKDAAENSLCAYKKA-AEDAEKLPVT
SMP4            LPNASPD--DSKVFFLKMQGDYYRYLAEVATDDARTEVVQKSLDAYTKA-TTAAENLPTT
SMP6            LVQESND--ESKVFFLKMQGDYNRYLAEVASDKTRAEVVQRSLDAYTKA-TEAANKLPTT
hsEP            IPAANTG--ESKVFYYKMKGDYHRYLAEFATGNDRKEAAENSLVAYKAA-SIAMTELPPT
hsS             IKEAGDA--ESRVFYLKMKGDYYRYLAEVATGDDKKRIIDSARSAYQEAMDISKKEMPPT
hsG             IKNCSETQYESKVFYLKMKGDYYRYLAEVATGEKRATVVESSEKAYSEAHEISKEHMQPT
hsET            IKNCNDFQYESKVFYLKMKGDYYRYLAEVASGEKKNSVVEASEAAYKEAFEISKEQMQPT
hsT             IANATNP--ESKVFYLKMKGDYFRYLAEVACGDDRKQTIDNSQGAYQEAFDISKKEMQPT
hsZ             IPNASQA--ESKVFYLKMKGDYYRYLAEVAAGDDKKGIVDQSQQAYQEAFEISKKEMQPT
hsA             IPNATQP--ESKVFYLKMKGDYFRYLSEVASGDNKQTTVSNSQQAYQEAFEISKKEMQPT
                         :  :      .:   :  :   .                        :
```

Supplemental Fig 2: Sequence Alignment of S. mansoni vs Human Isoforms

Supplemental Fig 2: cont'd

```
SMP5      HQVRLFQLGECKRLVSISYGVKQTLKRFSSVLNSDSDIISFSGHTLAEDARN--------
SMP3      HPIRL---GLALNFSVFYYEIENNPEKACSIAQTAFNESIGQLDQPDSGSFKDSTLVMQL
Smp2      HPIRL---GLALNYSVYFYEIMNDPDEACRLAQAAFDDAIAKLDQLSEESYKDSTLIMQL
Smp1      HPIRL---GLALNFSVFYYEILNNSEQACKCARVAFDSAIAELDTLSEESYKDSTIIMQL
SMP4      HPIRL---GLALNFSVFFYEIQNDAAKACELAKSAFDSAIAELDQLQDDSYKDSTLIMQL
SMP6      HPIRL---GLALNFSVFYYEIQNNAPQACELAKSAFDSAIAELDQLQDDSYKDSTLIMQL
hsEP      HPIRL---GLALNFSVFYYEILNSPDRACRLAKAAFDDAIAELDTLSEESYKDSTLIMQL
hsS       NPIRL---GLALNFSVFHYEIANSPEEAISLAKTTFDEAMADLHTLSEDSYKDSTLIMQL
hsG       HPIRL---GLALNYSVFYYEIQNAPEQACHLAKTAFDDAIAELDTLNEDSYKDSTLIMQL
hsET      HPIRL---GLALNFSVFYYEIQNAPEQACLLAKQAFDDAIAELDTLNEDSYKDSTLIMQL
hsT       HPIRL---GLALNFSVFYYEILNNPELACTLAKTAFDEAIAELDTLNEDSYKDSTLIMQL
hsZ       HPIRL---GLALNFSVFYYEILNSPEKACSLAKTAFDEAIAELDTLSEESYKDSTLIMQL
hsA       HPIRL---GLALNFSVFYYEILNSPEKACSLAKTAFDEAIAELDTLNEESYKDSTLIMQL
          : :**   * . .     * : :         .   :    .     . : :

SMP5      IRTRP---------------------------
SMP3      LRDNLTLWTSEREAAQ----------------
Smp2      LRDNLTLWTSDPERDDNVKKD------TDEKA
Smp1      LRDNLTLWTSNSEGEKDTASP------KGDKK
SMP4      LRDNLTLWASDQTAEGDVEND------S----
SMP6      LRDNLT-WASDQTAEGDGNS------------
hsEP      LRDNLTLWTSDMQGDGEEQNKEALQDVEDENQ
hsS       LRDNLTLWTADNAGEEGGEAP------QEPQS
hsG       LRDNLTLWTSDQQDDDGGEGN------N----
hsET      LRDNLTLWTSDQQDEEAGEGN-----------
hsT       LRDNLTLWTSDSAGEECDAAE------GAEN-
hsZ       LRDNLTLWTSDTQGDEAEAGE------GGEN-
hsA       LRDNLTLWTSENQGDEGDAGE-------GEN-
          .*
```
