# Supplementary material for: Bioinformatic analysis reveals new determinants of antigenic 14-3-3 proteins and a novel antifungal strategy
Source: PLoS One. 2017 Dec 12;12(12):e0189503. doi: 10.1371/journal.pone.0189503 (PMC5726717; doi:10.1371/journal.pone.0189503)
Supplement: S3 Fig — Alignment of A. cantonensis 14-3-3 with human zeta isoform showed significant homology between the two proteins. (PDF) [file pone.0189503.s003.pdf]

Supplemental Fig 3: Sequence Alignment of Angiostrongylus vs Human Isoforms

```
Angiostrongylus      MTDNRGELVQRAKLAEQAERYDDMAQSMKKVTELGAELSNEERNLLSVAYKNVVGARRSS 60
Human                MDKN--ELVQKAKLAEQAERYDDMAACMKSVTEQGAELSNEERNLLSVAYKNVVGARRSS 58
                     * .*  ****:************** .**.*** **************************

Angiostrongylus      WRVISSIEQKTEGSEKKQQMAKEYREKVEKELRDICQDVLNLLDKFLIPKAGNPESKVFY 120
Human                WRVVSSIEQKTEGAEKKQQMAREYREKIETELRDICNDVLSLLEKFLIPNASQAESKVFY 118
                     ***:*********:*******:*****:*.******:***.**:*****:*.:.******

Angiostrongylus      LKMKGDYYRYLAEVACGEDRSSVVEKSQQSYQEAFDIAKDKMQPTHPIRLGLALNFSVFY 180
Human                LKMKGDYYRYLAEVAAGDDKKGIVDQSQQAYQEAFEISKKEMQPTHPIRLGLALNFSVFY 178
                     ***************.*:*:..:*::***:*****:*:*.:*******************

Angiostrongylus      YEILNAPDKACQLAKQAFDDAIAELDTLNEDSYKDSTLIMQLLRDNLTLWTSDAAADDQD 240
Human                YEILNSPEKACSLAKTAFDEAIAELDTLSEESYKDSTLIMQLLRDNLTLWTSDTQGDEAE 238
                     *****:*:***.*** ***:********.*:**********************: .*: :

Angiostrongylus      TGEQGEGAN 249
Human                AGEGGEN-- 245
                     :** **.
```
