# Supplementary figures and images for: Bioinformatic analysis reveals new determinants of antigenic 14-3-3 proteins and a novel antifungal strategy
Source: PLoS One. 2017 Dec 12;12(12):e0189503. doi: 10.1371/journal.pone.0189503 (PMC5726717; doi:10.1371/journal.pone.0189503)

## Supplemental Fig 4: Effect of 14-3-3 Inhibitor on The Yeast Growth

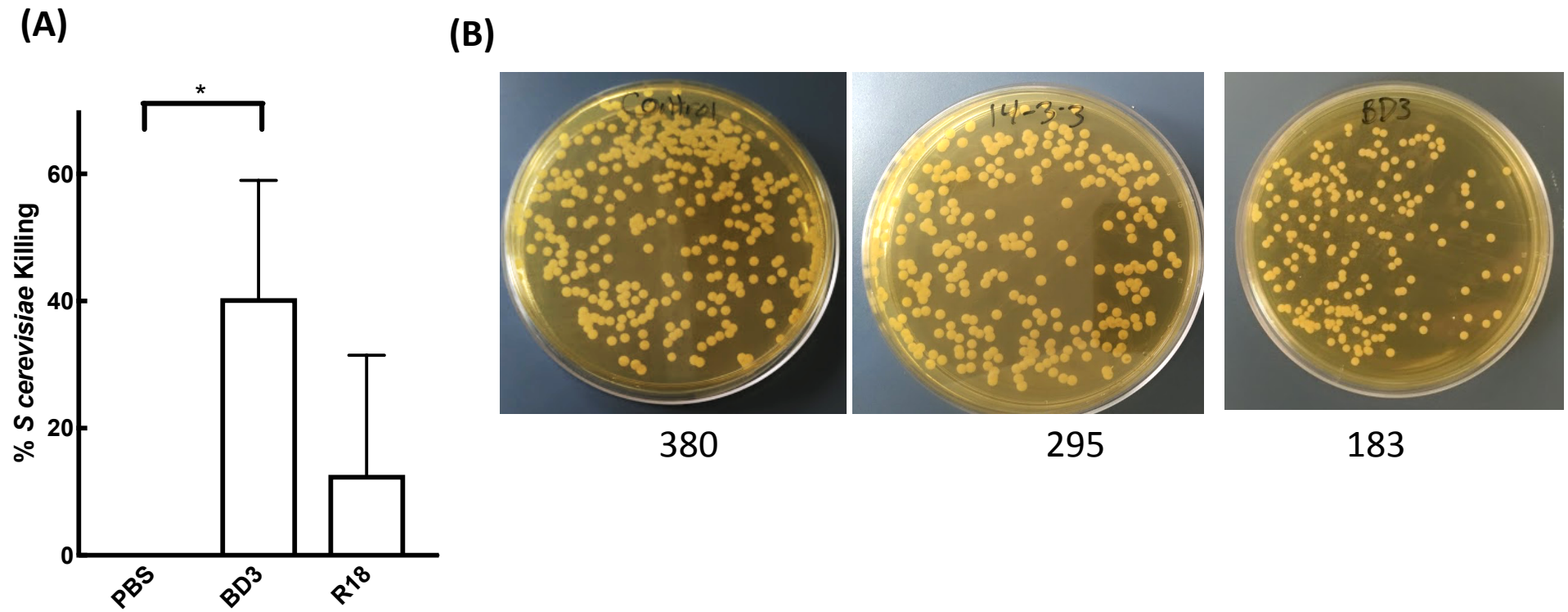

Supplement: S4 Fig — (A) Inhibition of 14-3-3 in S. cerevisiae does not decrease cell survivality. Unlike C. albicans, Inhibition of 14-3-3 by peptide inhibitor R18 (500nM) did not result in statistically significant change in the yeast survival in comparison to b-defensin 3 (BD3, 200nM). (B) Representative Images from the Candida survival experiment (Fig 7A) are shown. Number of counted colonies on each plate are listed below. (PDF) [file pone.0189503.s004.pdf]
