# Supplementary material for: Bioinformatic analysis reveals new determinants of antigenic 14-3-3 proteins and a novel antifungal strategy
Source: PLoS One. 2017 Dec 12;12(12):e0189503. doi: 10.1371/journal.pone.0189503 (PMC5726717; doi:10.1371/journal.pone.0189503)
Supplement: S5 Fig — A table showing abbreviations that are used in alignment file, followed by alignment file. (PDF) [file pone.0189503.s005.pdf]

# Supplemental Fig 5: Alignment of All 14-3-3 Genes Investigated in The Current Study

| Species | Gene | Abbreviation | Species | Gene | Abbreviation | Species | Gene | Abbreviation | Species | Gene | Abbreviation |
|---|---|---|---|---|---|---|---|---|---|---|---|
| Homo sapiens | YWHAZ | HsZ | Gallus gallus | SFN | GgS | Danio rerio | ywhag1 | DrG | Toxoplasma gondii- tg1 | TGME49_263090 | Tg1 |
| Homo sapiens | YWHAQ | HsT | Gallus gallus | YWHAZ | GgZ | Danio rerio | ywhaqa | DrT | Toxoplasma gondii- tg2 | TGME49_269960 | Tg2 |
| Homo sapiens | SFN | HsS | Gallus gallus | YWHAE | GgEP | Danio rerio | ywhaqb | DrTB | Schistosoma mansoni | Smp_041430.1 | Sm1 |
| Homo sapiens | YWHAE | HsEP | Gallus gallus | YWHAG | GgG | Danio rerio | ywhah | DrET | Schistosoma mansoni | Smp_009760 | Sm2 |
| Homo sapiens | YWHAB | HsA | Gallus gallus | YWHAQ | GgT | Danio rerio | ywhag2 | DrG1 | Schistosoma mansoni | Smp_009780.1 | Sm3 |
| Homo sapiens | YWHAG | HsG | Gallus gallus | YWHAB | GgA | Danio rerio | ywhae2 | DrEP2 | Schistosoma mansoni | Smp_002410 | Sm4 |
| Homo sapiens | YWHAH | HsET | Gallus gallus | YWHAH | GgET | Danio rerio | ywhaz | DrZ | Schistosoma mansoni | Smp_034840.1 | Sm5 |
| Mus musculus | Ywhaz | MmZ | Xenopus tropicalis | ywhaq | XtT | Drosophila melanogaster | 14-3-3zeta | DmZ | Ciona intestinalis | ci14-3-3 | Ci |
| Mus musculus | Sfn | MmS | Xenopus tropicalis | ywhag | XtG | Drosophila melanogaster | 14-3-3epsilon | DmEP | Oryza sativa- E | LOC4329775 | OsE |
| Mus musculus | Ywhae | MmEP | Xenopus tropicalis | ywhah | XtET | Saccharomyces cerevisiae | BMH1 | Sc1 | Oryza sativa-C | LOC4345634 | OsC |
| Mus musculus | Ywhaq | MmT | Xenopus tropicalis | ywhab | XtA | Saccharomyces cerevisiae | BMH2 | Sc2 | Oryza sativa- A | LOC4345850 | OsA |
| Mus musculus | Ywhab | MmA | Xenopus tropicalis | ywhae | XtEP | Candida albicans | BMH1 | Ca | Oryza sativa- D | LOC4350697 | OsD |
| Mus musculus | Ywhag | MmG | Xenopus tropicalis | ywhaz | XtZ | Schizosaccharomyces pombe | rad24 | Sp24 | Oryza sativa- B | LOC4336066 | OsB |
| Mus musculus | Ywhah | MmET | Danio rerio | ywhae1 | DrEP | Schizosaccharomyces pombe | rad25 | Sp25 | Oryza sativa- F | LOC4333880 | OsF |
| Danio rerio | ywhaba | DrAA | Danio rerio | ywhabb | DrAB | Oryza sativa- H | LOC9269633 | OsH | Oryza sativa- G | LOC4325534 | OsG |

CLUSTAL multiple sequence alignment by MUSCLE (3.8)

```
Tg3             -MSISLATLPLAGGLAVPPQEKTQRSQSGALRVPSSPPTPKADPSTPEHTSAFEKLKIHQ
Tg2             MASLPAPLSPNSGSSPPSPSTASLPFGIQLPLFDSAVSRPVSGGDAHRHTSTARIFPVLS
OsH             ------------------------------------------------------------
Sm1             ------------------------------------------------------------
Sm5             ------------------------------------------------------------
Sm2             ------------------------------------------------------------
Sm3             ------------------------------------------------------------
OsG             ------------------------------------------------------------
Tg1             ------------------------------------------------------------
Sm4             ------------------------------------------------------------
Sp25            ------------------------------------------------------------
Sp24            ------------------------------------------------------------
OsA             ------------------------------------------------------------
OsD             ------------------------------------------------------------
OsF             ------------------------------------------------------------
OsC             ------------------------------------------------------------
OsE             ------------------------------------------------------------
OsB             ------------------------------------------------------------
Ci              ------------------------------------------------------------
DmEP            ------------------------------------------------------------
DrEP2           ------------------------------------------------------------
XtEP            ------------------------------------------------------------
DrEP            ------------------------------------------------------------
HsEP            ------------------------------------------------------------
MmEP            ------------------------------------------------------------
GgEP            ------------------------------------------------------------
Ca              ------------------------------------------------------------
Sc1             ------------------------------------------------------------
Sc2             ------------------------------------------------------------
GgS             ------------------------------------------------------------
HsS             ------------------------------------------------------------
MmS             ------------------------------------------------------------
DrET            ------------------------------------------------------------
XtG             ------------------------------------------------------------
HsG             ------------------------------------------------------------
MmG             ------------------------------------------------------------
GgG             ------------------------------------------------------------
DrG             ------------------------------------------------------------
DrG1            ------------------------------------------------------------
XtET            ------------------------------------------------------------
GgET            ------------------------------------------------------------
HsET            ------------------------------------------------------------
MmET            ------------------------------------------------------------
XtT             ------------------------------------------------------------
DrZ             ------------------------------------------------------------
DmZ             ------------------------------------------------------------
GgT             ------------------------------------------------------------
HsT             ------------------------------------------------------------
MmT             ------------------------------------------------------------
DrT             ------------------------------------------------------------
DrTB            ------------------------------------------------------------
XtZ             ------------------------------------------------------------
GgZ             ------------------------------------------------------------
HsZ             ------------------------------------------------------------
```

```
MmZ        ------------------------------------------------------------
XtA        ------------------------------------------------------------
GgA        ------------------------------------------------------------
HsA        ------------------------------------------------------------
MmA        ------------------------------------------------------------
DrAB       ------------------------------------------------------------
DrAA       ------------------------------------------------------------


Tg3        IAMRVVKVAELQRRLRHKKLAPLQDEEQIAALSGIRLENVLAFRNLVSATKRATAASVPI
Tg2        SLAGAARNAALSPAHSEQRAAVSLGAQEADGDKWKQTLFEGNGGSPAEPRKGFLPDAHLL
OsH        ------------------------------------------------------------
Sm1        ------------------------------------------------------------
Sm5        ------------------------------------------------------------
Sm2        ------------------------------------------------------------
Sm3        ------------------------------------------------------------
OsG        ------------------------------------------------------------
Tg1        ------------------------MVSTDIAFSSAKKSTRRPLLVFHRPARDFFVFLRFP
Sm4        ------------------------------------------------------------
Sp25       ------------------------------------------------------------
Sp24       ------------------------------------------------------------
OsA        ------------------------------------------------------------
OsD        ------------------------------------------------------------
OsF        ------------------------------------------------------------
OsC        ------------------------------------------------------------
OsE        ------------------------------------------------------------
OsB        ------------------------------------------------------------
Ci         ------------------------------------------------------------
DmEP       ------------------------------------------------------------
DrEP2      ------------------------------------------------------------
XtEP       ------------------------------------------------------------
DrEP       ------------------------------------------------------------
HsEP       ------------------------------------------------------------
MmEP       ------------------------------------------------------------
GgEP       ------------------------------------------------------------
Ca         ------------------------------------------------------------
Sc1        ------------------------------------------------------------
Sc2        ------------------------------------------------------------
GgS        ------------------------------------------------------------
HsS        ------------------------------------------------------------
MmS        ------------------------------------------------------------
DrET       ------------------------------------------------------------
XtG        ------------------------------------------------------------
HsG        ------------------------------------------------------------
MmG        ------------------------------------------------------------
GgG        ------------------------------------------------------------
DrG        ------------------------------------------------------------
DrG1       ------------------------------------------------------------
XtET       ------------------------------------------------------------
GgET       ------------------------------------------------------------
HsET       ------------------------------------------------------------
MmET       ------------------------------------------------------------
XtT        ------------------------------------------------------------
DrZ        ------------------------------------------------------------
DmZ        ------------------------------------------------------------
GgT        ------------------------------------------------------------
HsT        ------------------------------------------------------------
```

```
MmT             ------------------------------------------------------------
DrT             ------------------------------------------------------------
DrTB            ------------------------------------------------------------
XtZ             ------------------------------------------------------------
GgZ             ------------------------------------------------------------
HsZ             ------------------------------------------------------------
MmZ             ------------------------------------------------------------
XtA             ------------------------------------------------------------
GgA             ------------------------------------------------------------
HsA             ------------------------------------------------------------
MmA             ------------------------------------------------------------
DrAB            ------------------------------------------------------------
DrAA            ------------------------------------------------------------

Tg3             GEKDPLLGRTTLALSIFNIPTEVQRRITDKDRWRRLFPAARGERPGGGLTLLFLALMAEA
Tg2             LAPTASTSFVPPLVSFQEKEAPQTRAAEDLAH------------------LQAQASIAAN
OsH             --------------------------MKEREK------------------VVRLAKLAEQ
Sm1             ---------------MCDDSWLNESSIKDKDS------------------LITLAKIQEQ
Sm5             ------------------------------------------------------------
Sm2             ----------------MTTSWVLQSKDLSNTD------------------LVHIAKLAEQ
Sm3             ----------------MTTSWVTQCEDLSNTD------------------LVHIAKLAEQ
OsG             --------------------------MAPSDD------------------LVYMAKLAEQ
Tg1             SFFSGHFPSFPLLLSCVPVSTMAEEIKNLRDE------------------YVYKAKLAEQ
Sm4             -------------------------MDELRER------------------NIVLAKLCEQ
Sp25            -------------------------MSNSREN------------------SVYLAKLAEQ
Sp24            ------------------------MSTTSRED------------------AVYLAKLAEQ
OsA             --------------------MAAAAGGGTREE------------------MVYMAKLAEQ
OsD             ----------------------MSPAEPTREE------------------SVYKAKLAEQ
OsF             ----------------------MSPAEASREE------------------NVYMAKLAEQ
OsC             ---------------------------MSREE------------------NVYMAKLAEQ
OsE             ---------MENYVRKTQTVKMSQPAELSREE------------------NVYMAKLAEQ
OsB             ---------------------MSAQAELSREE------------------NVYMAKLAEQ
Ci              -------------------------MSTERED------------------FVYQAKLAEQ
DmEP            --------------------------MTEREN------------------NVYKAKLAEQ
DrEP2           --------------------------MADREH------------------LVYQAKLAEQ
XtEP            --------------------------MEERED------------------LVYRAKLAEQ
DrEP            --------------------------MGDRED------------------LVYQAKLAEQ
HsEP            --------------------------MDDRED------------------LVYQAKLAEQ
MmEP            --------------------------MDDRED------------------LVYQAKLAEQ
GgEP            --------------------------MDDRED------------------LVYQAKLAEQ
Ca              -------------------------MPASRED------------------SVYLAKLAEQ
Sc1             -------------------------MSTSRED------------------SVYLAKLAEQ
Sc2             -------------------------MSQTRED------------------SVYLAKLAEQ
GgS             ---------------------------MARNH------------------QVQKAKLAEQ
HsS             ---------------------------MERAS------------------LIQKAKLAEQ
MmS             ---------------------------MERAS------------------LIQKAKLAEQ
DrET            --------------------------MADREQ------------------LIQRARLAEQ
XtG             --------------------------MVDREQ------------------LVQKARLAEQ
HsG             --------------------------MVDREQ------------------LVQKARLAEQ
MmG             --------------------------MVDREQ------------------LVQKARLAEQ
GgG             --------------------------MVDREQ------------------LVQKARLAEQ
DrG             --------------------------MVDREQ------------------LVQKARLAEQ
DrG1            --------------------------MVDREQ------------------LVQKARLAEQ
XtET            --------------------------MADREQ------------------LLQRARLAEQ
GgET            --------------------------MGDREQ------------------LLQRARLAEQ
HsET            --------------------------MGDREQ------------------LLQRARLAEQ
```

```
MmET      --------------------------MGDREQ------------------LLQRARLAEQ
XtT       ---------------------------MDRNA------------------QIQKAKLAEQ
DrZ       ---------------------------MDKSQ------------------HVQRAKLAEQ
DmZ       ------------------------MSTVDKEE------------------LVQKAKLAEQ
GgT       ---------------------------MDKTE------------------LIQKAKLAEQ
HsT       ---------------------------MEKTE------------------LIQKAKLAEQ
MmT       ---------------------------MEKTE------------------LIQKAKLAEQ
DrT       ---------------------------MDKLE------------------LIQKAKLAEQ
DrTB      ---------------------------MDRTE------------------LIQKAKLAEQ
XtZ       ---------------------------MDKNE------------------LVQKAKLAEQ
GgZ       ---------------------------MDKNE------------------LVQKAKLAEQ
HsZ       ---------------------------MDKNE------------------LVQKAKLAEQ
MmZ       ---------------------------MDKNE------------------LVQKAKLAEQ
XtA       ---------------------------MDKSE------------------LVQKAKLSEQ
GgA       ---------------------------MDKSE------------------LVQKAKLAEQ
HsA       -------------------------MTMDKSE------------------LVQKAKLAEQ
MmA       -------------------------MTMDKSE------------------LVQKAKLAEQ
DrAB      ---------------------------MDKSD------------------LVQKAKLAEQ
DrAA      ---------------------------MDKSD------------------LVQKAKLAEQ


Tg3       CRRFDEADVYVIQLLVRKAS------R-LEELTEEERACVQRAFNRRI------------
Tg2       LENWQD----VIRVMKKLAQ------V-HPNLDSTQRSLVVDAYTNLANEACAARKTLDG
OsH       AERYDD----MVEFMKTLAR------M-DVDMSAEERLLFSVGFKKTI------------
Sm1       AERFND----MACAMKKVVE------T-SKTLNNEERNLFSVAYKNVV------------
Sm5       ----------MVDAMKEVVE------M-AEELTVEERNLLSVAYKNVI------------
Sm2       AERYDD----MAAAMKRYTE------A-SGNLGNEERNLLSVAYKNVV------------
Sm3       AERYDD----MAAAMKRYTE------A-SGTLGNEERNLLSVAYKNVV------------
OsG       AERYDE----MVEAMNSVAK------L-DEGLTKEERNLLSVGYKNLI------------
Tg1       AERYDE----MAEAMKNLVENCLDEQQPKDELSVEERNLLSVAYKNAV------------
Sm4       AERYDE----MVKAMIEIATN-----T-ETELTVEERNLLSVAYKNVI------------
Sp25      AERYEE----MVENMKKVAC------S-NDKLSVEERNLLSVAYKNII------------
Sp24      AERYEG----MVENMKSVAS------T-DQELTVEERNLLSVAYKNVI------------
OsA       AERYEE----MVEFMEKVVTAA-AAGG-GGELTVEERNLLSVAYKNVI------------
OsD       AERYEE----MVEYMERVARAAGGASG-GEELTVEERNLLSVAYKNVI------------
OsF       AERYEE----MVEFMEKVAKTT----D-VGELTVEERNLLSVAYKNVI------------
OsC       AERYEE----MVEYMEKVAKTV----D-VEELTVEERNLLSVAYKNVI------------
OsE       AERYEE----MVEFMEKVAKTV----D-SEELTVEERNLLSVAYKNVI------------
OsB       AERYEE----MVEFMEKVAKTV----D-SEELTVEERNLLSVAYKNVI------------
Ci        AERYDE----MVEQMKKIAQ------L-GISLTVEERNLLSVAYKNVI------------
DmEP      AERYDE----MVEAMKKVAS------M-DVELTVEERNLLSVAYKNVI------------
DrEP2     AERYDE----MVESMKNVAG------K-DEDLSVEERNLLSVAYKNVI------------
XtEP      AERYDE----MVESMKKVAG------M-DVELTVEERNLLSVAYKNVI------------
DrEP      AERYDE----MVDSMKKVAG------M-DVELTVEERNLLSVAYKNVI------------
HsEP      AERYDE----MVESMKKVAG------M-DVELTVEERNLLSVAYKNVI------------
MmEP      AERYDE----MVESMKKVAG------M-DVELTVEERNLLSVAYKNVI------------
GgEP      AERYDE----MVESMKKVAG------M-DVELTVEERNLLSVAYKNVI------------
Ca        AERYEE----MVENMKAVAS------S-GQELSVEERNLLSVAYKNVI------------
Sc1       AERYEE----MVENMKTVAS------S-GQELSVEERNLLSVAYKNVI------------
Sc2       AERYEE----MVENMKAVAS------S-GQELSVEERNLLSVAYKNVI------------
GgS       AERYED----MADFMKAVVE------H-GDELSNEERNLLSVAYKNVV------------
HsS       AERYED----MAAFMKGAVE------K-GEELSCEERNLLSVAYKNVV------------
MmS       AERYED----MAAFMKSAVE------K-GEELSCEERNLLSVAYKNVV------------
DrET      AERYDD----MASAMKLVTE------L-NEPLSNEDRNLLSVAYKNVV------------
XtG       AERYDD----MAAAMKAVTE------L-NEPLSNEERNLLSVAYKNVV------------
HsG       AERYDD----MAAAMKNVTE------L-NEPLSNEERNLLSVAYKNVV------------
MmG       AERYDD----MAAAMKNVTE------L-NEPLSNEERNLLSVAYKNVV------------
```

```
GgG             AERYDD----MAAAMKNVTE------L-NEPLSNEERNLLSVAYKNVV------------
DrG             AERYDD----MAAAMKSVTE------L-NEALSNEERNLLSVAYKNVV------------
DrG1            AERYDD----MAAAMKSVTE------L-NEALSNEERNLLSVAYKNVV------------
XtET            AERYED----MAAAMKSVTE------L-NEPLSNEDRNLLSVAYKNVV------------
GgET            AERYDD----MASAMKSVTE------L-NEPLSNEDRNLLSVAYKNVV------------
HsET            AERYDD----MASAMKAVTE------L-NEPLSNEDRNLLSVAYKNVV------------
MmET            AERYDD----MASAMKAVTE------L-NEPLSNEDRNLLSVAYKNVV------------
XtT             AERYDD----MAASMKAVTE------C-GTELSSEERNLLSVAYKNVV------------
DrZ             AERYDD----MADSMKKVTE------L-GEELSDEERNLLSVAYKNVV------------
DmZ             SERYDD----MAQAMKSVTE------T-GVELSNEERNLLSVAYKNVV------------
GgT             AERYDD----MATCMKAVTE------Q-GAELSNEERNLLSVAYKNVV------------
HsT             AERYDD----MATCMKAVTE------Q-GAELSNEERNLLSVAYKNVV------------
MmT             AERYDD----MATCMKAVTE------Q-GAELSNEERNLLSVAYKNVV------------
DrT             AERYDD----MAACMKQVTE------Q-GEELSNEERNLLSVAYKNVV------------
DrTB            AERYDD----MASCMKSVTE------A-GSELSNEERNLLSVAYKNVV------------
XtZ             AERYDD----MAACMKRVTE------E-GGELSNEERNLLSVAYKNVV------------
GgZ             AERYDD----MASCMKSVTE------Q-GAELSNEERNLLSVAYKNVV------------
HsZ             AERYDD----MAACMKSVTE------Q-GAELSNEERNLLSVAYKNVV------------
MmZ             AERYDD----MAACMKSVTE------Q-GAELSNEERNLLSVAYKNVV------------
XtA             AERYDD----MAASMKAVTE------L-GAELSNEERNLLSVAYKNVV------------
GgA             AERYDD----MAAAMKAVTE------Q-GHELSNEERNLLSVAYKNVV------------
HsA             AERYDD----MAAAMKAVTE------Q-GHELSNEERNLLSVAYKNVV------------
MmA             AERYDD----MAAAMKAVTE------Q-GHELSNEERNLLSVAYKNVV------------
DrAB            AERYDD----MAAAMKAVTE------G-GVELSNEERNLLSVAYKNVV------------
DrAA            AERYDD----MAASMKAVTE------G-GVELSNEERNLLSVAYKNVV------------
                          :   :   .            :   :*  .  .:..

Tg3             -------------------QRSKHSLKQVTALERAFDARRDAGQPNR-------------
Tg2             CSSALAALSAEPACSPEEQGAEKSA--VAESEEQSLSRDG-EEKQTREEAALSTLKTLGL
OsH             -------------------GARRASWRILESLEQKV--TA-GDQPGV-------------
Sm1             -------------------GCCRSAWRVVSNIEQRL-----DDQKKK-------------
Sm5             -------------------GSRRSSWRVFSAVEQTEGNRG-NAEKQA-------------
Sm2             -------------------GARRSAWRVIHGSEMKAVNDR---TKKQ-------------
Sm3             -------------------GARRSAWRVISGSETKAANDH---MKSQ-------------
OsG             -------------------GAKRAAMRIIGSIELKEETKG-KESHVR-------------
Tg1             -------------------GARRASWRIISSVEQKELSKQ-HMQNKA-------------
Sm4             -------------------GARRSSWRIINSKESQDEAKG--SDEIH-------------
Sp25            -------------------GARRASWRIISSIEQKEESRG-NTRQAA-------------
Sp24            -------------------GARRASWRIVSSIEQKEESKG-NTAQVE-------------
OsA             -------------------GARRASWRIVSSIEQKEEGRG-AAGHAA-------------
OsD             -------------------GARRASWRIISSIEQKEEGRG-NDAHAA-------------
OsF             -------------------GARRASWRIISSIEQKEESRG-NEAYVA-------------
OsC             -------------------GARRASWRIVSSIEQKEEGRG-NEEHVT-------------
OsE             -------------------GARRASWRIISSIEQKEESRG-NEDRCT-------------
OsB             -------------------GARRASWRIISSIEQKEESRG-NEDRVT-------------
Ci              -------------------GARRASWRIISSIEMK---EG---SKAQ-------------
DmEP            -------------------GARRASWRIITSIEQKEENKG-AEEKLE-------------
DrEP2           -------------------GARRASWRIISSIEQKEESKG-GADKLK-------------
XtEP            -------------------GARRASWRIISSIEQKEENKG-GEDKLK-------------
DrEP            -------------------GARRASWRIISSIEQKEENKG-GEDKLK-------------
HsEP            -------------------GARRASWRIISSIEQKEENKG-GEDKLK-------------
MmEP            -------------------GARRASWRIISSIEQKEENKG-GEDKLK-------------
GgEP            -------------------GARRASWRIISSIEQKEENKG-GEDKLK-------------
Ca              -------------------GARRASWRIVSSIEQKEEAKG-NESQVA-------------
Sc1             -------------------GARRASWRIVSSIEQKEESKEKSEHQVE-------------
Sc2             -------------------GARRASWRIVSSIEQKEESKEKSEHQVE-------------
GgS             -------------------GCQRSAWRVISSIEHKT--EE-GDDKAQ-------------
```

```
HsS             --------------------GGQRAAWRVLSSIEQKSNEEG-SEEKGP-------------
MmS             --------------------GGQRAAWRVLSSIEQKSNEEG-SEEKGP-------------
DrET            --------------------GARRSSWRVISSIEQKTAADG-NEKKLE-------------
XtG             --------------------GARRSSWRVISSIEQKTSADG-NEKKIE-------------
HsG             --------------------GARRSSWRVISSIEQKTSADG-NEKKIE-------------
MmG             --------------------GARRSSWRVISSIEQKTSADG-NEKKIE-------------
GgG             --------------------GARRSSWRVISSIEQKTSADG-NEKKIE-------------
DrG             --------------------GARRSSWRVISSIEQKTSADG-NEKKIE-------------
DrG1            --------------------GARRSSWRVISSIEQKTSADG-NEKKIE-------------
XtET            --------------------GARRSSWRVISSIEQKTLADG-NEKKLE-------------
GgET            --------------------GARRSSWRVISSIEQKTMADG-NEKKLE-------------
HsET            --------------------GARRSSWRVISSIEQKTMADG-NEKKLE-------------
MmET            --------------------GARRSSWRVISSIEQKTMADG-NEKKLE-------------
XtT             --------------------GARRSAWRVISSIEQKS--DS-EETKLK-------------
DrZ             --------------------GARRSAWRVVSSIEQKT--EG-TDKK-Q-------------
DmZ             --------------------GARRSSWRVISSIEQKT--EA-SARKQQ-------------
GgT             --------------------GGRRSAWRVISSIEQKT--DT-SDKKMQ-------------
HsT             --------------------GGRRSAWRVISSIEQKT--DT-SDKKLQ-------------
MmT             --------------------GGRRSAWRVISSIEQKT--DT-SDKKLQ-------------
DrT             --------------------GARRSAWRVISSIEQKT--EG-NDKKLQ-------------
DrTB            --------------------GARRSAWRVISSIEQKT--EG-NDKKLQ-------------
XtZ             --------------------GARRSSWRVVSSIEQKT--EG-AEKKQE-------------
GgZ             --------------------GARRSSWRVVSSIEQKT--EG-AEKKQQ-------------
HsZ             --------------------GARRSSWRVVSSIEQKT--EG-AEKKQQ-------------
MmZ             --------------------GARRSSWRVVSSIEQKT--EG-AEKKQQ-------------
XtA             --------------------GARRSSWRVISSIEQKT--EG-NDKRQQ-------------
GgA             --------------------GARRSSWRVISSIEQKT--ER-NEKKQQ-------------
HsA             --------------------GARRSSWRVISSIEQKT--ER-NEKKQQ-------------
MmA             --------------------GARRSSWRVISSIEQKT--ER-NEKKQQ-------------
DrAB            --------------------GARRSSWRVISSIEQKT--EG-NEKKQQ-------------
DrAA            --------------------GARRSSWRVISSIEQKT--EG-NEKKQQ-------------
                                      . :       *

Tg3             ------------TYRLAEVAAYKERLKDDIVRHCYAIVWAVTHLIMPSPGSA---ATQMF
Tg2             GLVSATQLHEFTAFFEFCVNLYKQRVTDDLFALNEDVDRFVLGVLLPQAENH---EATAA
OsH             -----------------TINGYKKKVEDELRAVCNEVLSIIAIHCLPLANSG---ENVVF
Sm1             -----------------QAGEYRSTIEKELQAVCQEVLDLLHESLLKSENTA---EGFVF
Sm5             -----------------CAKKFREVLESELDRVSKDILELIDKYLIKSATKS---DSKVF
Sm2             -----------------IAEEYRIKMEKELNTICNQVLALLEDYLLPNASPD---DSKVF
Sm3             -----------------IAEEYRIKIEKELNAICDQVLVLLKDYLLVQESND---ESKVF
OsG             -----------------QTAEYRRKVEAEMDKICCDVINIIDKYLIPHSSGA---ESSVF
Tg1             -----------------LAAEYRQKVEEELNKICHDILQLLTDKLIPKTSDS---ESKVF
Sm4             -----------------ITKRFRKEVEKELDEICTSILNLLDNCLLPKAVSD---ESKVF
Sp25            -----------------LIKEYRKKIEDELSDICHDVLSVLEKHLIPAATTG---ESKVF
Sp24            -----------------LIKEYRQKIEQELDTICQDILTVLEKHLIPNAASA---ESKVF
OsA             -----------------AARSYRARVEAELSNICAGILRLLDERLVPAAAAV---DAKVF
OsD             -----------------TIRSYRGKIEAELARICDGILALLDSHLVPSAGAA---ESKVF
OsF             -----------------SIKEYRSRIETELSKICDGILKLLDSHLVPSATAA---ESKVF
OsC             -----------------LIKEYRGKIEAELSKICDGILKLLDSHLVPSSTAA---ESKVF
OsE             -----------------LIKEYRGKIETELSKICDGILKLLDSHLVPSSTAP---ESKVF
OsB             -----------------LIKDYRGKIETELTKICDGILKLLESHLVPSSTAP---ESKVF
Ci              -----------------MPSDYRQQVEEELKNICHDILSVIDEHLLPPSQTDQDIESQVF
DmEP            -----------------MIKTYRGQVEKELRDICSDILNVLEKHLIPCATSG---ESKVF
DrEP2           -----------------MIREYRQTVENELKSICNDILDVLDKHLIPAANTG---ESKVF
XtEP            -----------------MIREYRQMVEAELKSICNDILDVLDKHLIPAANSG---ESKVF
DrEP            -----------------MIREYRQTVENELKSICNDILDVLDKHLIPAANSG---ESKVF
HsEP            -----------------MIREYRQMVETELKLICCDILDVLDKHLIPAANTG---ESKVF
```

```
MmEP        -----------------MIREYRQMVETELKLICCDILDVLDKHLIPAANTG---ESKVF
GgEP        -----------------MIREYRQMVETELKLICCDILDVLDKHLIPAANTG---ESKVF
Ca          -----------------LIRDYRAKIEAELSKICEDILSVLSDHLITSAQTG---ESKVF
Sc1         -----------------LICSYRSKIETELTKISDDILSVLDSHLIPSATTG---ESKVF
Sc2         -----------------LIRSYRSKIETELTKISDDILSVLDSHLIPSATTG---ESKVF
GgS         -----------------LVNEYREKVEEELKGVCNVVLGLLEKHLIKKAGDA---ESKVF
HsS         -----------------EVREYREKVETELQGVCDTVLGLLDSHLIKEAGDA---ESRVF
MmS         -----------------EVKEYREKVETELRGVCDTVLGLLDSHLIKGAGDA---ESRVF
DrET        -----------------LVRVYRETVEKELESVCQDVLTLLDQYLIKNCDETQV-ESKVF
XtG         -----------------MVRAYREKIEKELEAVCQDVLSLLDNFLIKNCSETQY-ESKVF
HsG         -----------------MVRAYREKIEKELEAVCQDVLSLLDNYLIKNCSETQY-ESKVF
MmG         -----------------MVRAYREKIEKELEAVCQDVLSLLDNYLIKNCSETQY-ESKVF
GgG         -----------------MVRAYREKIEKELGAVCQDVLSLLDNYLIKNCSETQY-ESKVF
DrG         -----------------MVRAYREKIEKELETVCQDVLNLLDNFLIKNCGETQH-ESKVF
DrG1        -----------------MVRAYREKIEKELEAVCQDVLNLLDNFLIKNCSETQH-ESKVF
XtET        -----------------KVKAYREKIEAELEAVCSEVLSLLDKFLIKNCNDFQY-ESKVF
GgET        -----------------KVKAYREKIEKELETVCNDVLALLDKYLIKNCNDFQY-ESKVF
HsET        -----------------KVKAYREKIEKELETVCNDVLSLLDKFLIKNCNDFQY-ESKVF
MmET        -----------------KVKAYREKIEKELETVCNDVLALLDKFLIKNCNDFQY-ESKVF
XtT         -----------------IAREYKEKVESELQNICETVLNLLDKHLISSSTAT---ESQVF
DrZ         -----------------MAQEYREKIEAELKAICNDVLHLLDKFLIRSTSPA---ESQVF
DmZ         -----------------LAREYRERVEKELREICYEVLGLLDKYLIPKASNP---ESKVF
GgT         -----------------LIKDYREKVESELRSICTTVLELLDKYLIANATNP---ESKVF
HsT         -----------------LIKDYREKVESELRSICTTVLELLDKYLIANATNP---ESKVF
MmT         -----------------LIKDYREKVESELRSICTTVLELLDKYLIANATNP---ESKVF
DrT         -----------------MVKEYREKVEGELRDICNEVLTLLGKYLIKNSTNS---ESKVF
DrTB        -----------------MVKEYREKVESELRDICNDVLELLNKYLIENSSNP---ESKVF
XtZ         -----------------MSREYREKIEAELREICNDVLNLLDKFLIANASQP---ESKVF
GgZ         -----------------MAREYREKIETELRDICNDVLSLLEKFLIPNASQA---ESKVF
HsZ         -----------------MAREYREKIETELRDICNDVLSLLEKFLIPNASQA---ESKVF
MmZ         -----------------MAREYREKIETELRDICNDVLSLLEKFLIPNASQP---ESKVF
XtA         -----------------MAREYREKVETELQDICKDVLGLLDKYLVPNATPP---ESKVF
GgA         -----------------MGREYREKIEAELQDICNDVLELLDKYLIVNATQP---ESKVF
HsA         -----------------MGKEYREKIEAELQDICNDVLELLDKYLIPNATQP---ESKVF
MmA         -----------------MGKEYREKIEAELQDICNDVLELLDKYLILNATQA---ESKVF
DrAB        -----------------MAREYREKIETELQDICSDVLGLLEKYLIANASQA---ESKVF
DrAA        -----------------MAREYREKIEAELQEICNDVLGLLEKYLIPNASQA---ESKVF
                                  :.  :  ::       :   :    :

Tg3         SYKWIATAFRQVTQFTDSVP-QYQCLQLAESNYQQAVNVAKTDTTLR-PCDMLRIETLIS
Tg2         YQQLRGDVSRHTAALTKNAEIRRRMEERALRAYESALQSTEQD--DELKVTPLHLGIVLN
OsH         FYKMKGDYYRYLAEFSTGTE-KKAATDQSLMAYQHAMVVASSE--LS-PAHQFRLGLALN
Sm1         YKKMEGDYYRYLAEVLTGDK-SADVVKHSREAYQAATEKANSD--LP-PTHPIRLGLALN
Sm5         YLKMKGDYFRYMAEFSVDPQ-RKKAAEESNKAYQEASEIAATQ--LF-PTHPIRLGLALN
Sm2         FLKMQGDYYRYLAEVATDDA-RTEVVQKSLDAYTKATT-AAEN--LP-TTHPIRLGLALN
Sm3         FLKMQGDYNRYLAEVASDKT-RAEVVQRSLDAYTKATEAANK---LP-TTHPIRLGLALN
OsG         YYKMKGDYYRYLAEFKTGTE-KIEVSELSLNAYETASKTAQTD--LT-PTDPIRLGLALN
Tg1         YYKMKGDYYRYISEFSGEEG-KKQAADQAQESYQKATETAEAE--LP-STHPIRLGLALN
Sm4         LNKMRGDYHRYRAEYSVGNQ-RKDAAENSLCAYKKAAEDAEK---LP-VTHPIRLGLALN
Sp25        YYKMKGDYYRYLAEFTVGEV-CKEAADSSLEAYKAASDIAVAE--LP-PTDPMRLGLALN
Sp24        YYKMKGDYYRYLAEFAVGEK-RQHSADQSLEGYKAASEIATAE--LA-PTHPIRLGLALN
OsA         YLKMKGDYHRYLAEFKTGAE-RKDAADATLAAYQAAQDIAMKE--LS-PTHPIRLGLALN
OsD         YLKMKGDYHRYLAEFKSGDE-RKQAAESTMNAYKAAQDIALAD--LA-PTHPIRLGLALN
OsF         YLKMKGDYHRYLAEFKSGAE-RKEAAENTLVAYKSAQDIALAD--LP-TTHPIRLGLALN
OsC         YLKMKGDYHRYLAEFKTGAE-RKEAAESTMVAYKAAQDIALAD--LA-PTHPIRLGLALN
OsE         YLKMKGDYYRYLAEFKTGAE-RKDAAENTMVAYKAAQDIALAE--LP-PTHPIRLGLALN
OsB         YLKMKGDYYRYLAEFKTGAE-RKDAAENTMVAYKAAQDIALAE--LP-PTHPIRLGLALN
```

```
Ci              YHKMKGDYHRYLAEFETGDE-RKSAAEDSLTAYKAASDAAGK---LP-TTHPIRLGLALN
DmEP            YYKMKGDYHRYLAEFATGSD-RKDAAENSLIAYKAASDIAMND--LP-PTHPIRLGLALN
DrEP2           YYKMKGDYHRYLAEFATGND-RKEAAENSLVAYKAASDIAMTE--LP-PTHPIRLGLALN
XtEP            YYKMKGDYHRYLAEFAQGND-RKEAAENSLVAYKAASDIAMTE--LP-PTHPIRLGLALN
DrEP            YYKMKGDYHRYLAEFATGND-RKEAAENSLVAYKAASDIAMTD--LQ-PTHPIRLGLALN
HsEP            YYKMKGDYHRYLAEFATGND-RKEAAENSLVAYKAASDIAMTE--LP-PTHPIRLGLALN
MmEP            YYKMKGDYHRYLAEFATGND-RKEAAENSLVAYKAASDIAMTE--LP-PTHPIRLGLALN
GgEP            YYKMKGDYHRYLAEFATGND-RKEAAENSLVAYKAASDIAMTE--LP-PTHPIRLGLALN
Ca              YYKMKGDYHRYLAEFAIAEK-RKEAADLSLEAYKAASDVAVTE--LP-PTHPIRLGLALN
Sc1             YYKMKGDYHRYLAEFSSGDA-REKATNASLEAYKTASEIATTE--LP-PTHPIRLGLALN
Sc2             YYKMKGDYHRYLAEFSSGDA-REKATNSSLEAYKTASEIATTE--LP-PTHPIRLGLALN
GgS             YLKMKGDYFRYLAEVAAGND-RKETIDSAQKAYQEAMDISKKE--MQ-PTNPIRLGLALN
HsS             YLKMKGDYYRYLAEVATGDD-KKRIIDSARSAYQEAMDISKKE--MP-PTNPIRLGLALN
MmS             YLKMKGDYYRYLAEVATGDD-KKRIIDSARSAYQEAMDISKKE--MP-PTNPIRLGLALN
DrET            YLKMKGDYYRYLAEVATGEK-RASAVESSEGAYKEAFDISKG---MP-ATHPIRLGLALN
XtG             YLKMKGDYYRYLAEVATGEK-RATVVESSEKAYSEAHEISKEH--MQ-PTHPIRLGLALN
HsG             YLKMKGDYYRYLAEVATGEK-RATVVESSEKAYSEAHEISKEH--MQ-PTHPIRLGLALN
MmG             YLKMKGDYYRYLAEVATGEK-RATVVESSEKAYSEAHEISKEH--MQ-PTHPIRLGLALN
GgG             YLKMKGDYYRYLAEVATGEK-RATVVESSEKAYSEAHEISKEH--MQ-PTHPIRLGLALN
DrG             YLKMKGDYYRYLAEVATGEK-RAAVVESSEKSYSEAHEISKEH--MQ-PTHPIRLGLALN
DrG1            YLKMKGDYYRYLAEVATGEK-RSTVVESSEKSYNEAHEISKEH--MQ-PTHPIRLGLALN
XtET            YLKMKGDYYRYLSEVGSGER-KRSVTEASEAAYKEAFEISKEH--MQ-PTHPIRLGLALN
GgET            YLKMKGDYYRYLAEVAAGEK-KNSVVEASEAAYKEAFEISKEH--MQ-PTHPIRLGLALN
HsET            YLKMKGDYYRYLAEVASGEK-KNSVVEASEAAYKEAFEISKEQ--MQ-PTHPIRLGLALN
MmET            YLKMKGDYYRYLAEVASGEK-KNSVVEASEAAYKEAFEISKEH--MQ-PTHPIRLGLALN
XtT             YLKMKGDYYRYLAEVATGDN-RTKTIQDSQAAYQEAFDISKKD--MQ-PTHPIRLGLALN
DrZ             YLKMKGDYYRYLAEVATGDE-KTNIIQKSQEGYQAAFDISKDN--MQ-PTHPIRLGLALN
DmZ             YLKMKGDYYRYLAEVATGDA-RNTVVDDSQTAYQDAFDISKGK--MQ-PTHPIRLGLALN
GgT             YLKMKGDYFRYLAEVACGDD-RKQTIENSQGAYQEAFDISKKE--MQ-PTHPIRLGLALN
HsT             YLKMKGDYFRYLAEVACGDD-RKQTIDNSQGAYQEAFDISKKE--MQ-PTHPIRLGLALN
MmT             YLKMKGDYFRYLAEVACGDD-RKQTIENSQGAYQEAFDISKKE--MQ-PTHPIRLGLALN
DrT             YLKMKGDYYRYLAEVAAADD-KMDTITNSQGAYQDAFEISKKD--MQ-PTHPIRLGLALN
DrTB            YLKMKGDYYRYLAEVAAGDD-KKATIENSQDAYQKAFDISKTE--MQ-PTHPIRLGLALN
XtZ             YLKMKGDYYRYLAEVASGDA-KADIVAQSQKAYQDAFDISKTE--MQ-PTHPIRLGLALN
GgZ             YLKMKGDYYRYLAEVAAGDD-KKGIVEQSQQAYQEAFEISKKE--MQ-PTHPIRLGLALN
HsZ             YLKMKGDYYRYLAEVAAGDD-KKGIVDQSQQAYQEAFEISKKE--MQ-PTHPIRLGLALN
MmZ             YLKMKGDYYRYLAEVAAGDD-KKGIVDQSQQAYQEAFEISKKE--MQ-PTHPIRLGLALN
XtA             YLKMKGDYYRYLSEVASGDS-KQETVTCSQQAYQEAFEISKSE--MQ-PTHPIRLGLALN
GgA             YLKMKGDYYRYLSEVASGDN-KQTTVANSQQAYQEAFEISKKE--MQ-PTHPIRLGLALN
HsA             YLKMKGDYFRYLSEVASGDN-KQTTVSNSQQAYQEAFEISKKE--MQ-PTHPIRLGLALN
MmA             YLKMKGDYFRYLSEVASGEN-KQTTVSNSQQAYQEAFEISKKE--MQ-PTHPIRLGLALN
DrAB            YLKMKGDYYRYLSEVASGDS-KATTVENSQKAYQDAFDISKKD--MQ-PTHPIRLGLALN
DrAA            YLKMKGDYYRYLSEVASGDS-KRTTVENSQKAYQDAFEISKKE--MQ-PTHPIRLGLALN
                  :  .   *  :               :   *  *   :            :.:   :.

Tg3             WANFLFYNLEKRQ--EALASARETLQEAIAQL-ETVPEEHFAEVVEALQILMKSVWRWNQ
Tg2             YGVLLKSINQGQQTNRAIELIAAAFRYSVENMYHVRNEEEYQRVLVILSLLRDNIEKWCA
OsH             FSVF-XEIMNSPE--RASQVAKQALDEATAEI-NSAGVEGYKDSMLMMQLLKENLALWTS
Sm1             FSVFYYEIENNPE--KACSIAQTAFNESIGQL-DQPDSGSFKDSTLVMQLLRDNLTLWTS
Sm5             YSVYFYEIMNDPD--EACRLAQAAFDDAIAKL-DQLSEESYKDSTLIMQLLRDNLTLWTS
Sm2             FSVFFYEIQNDAA--KACELAKSAFDSAIAEL-DQLQDDSYKDSTLIMQLLRDNLTLWAS
Sm3             FSVFYYEIQNNAP--QACELAKSAFDSAIAEL-DQLQDDSYKDSTLIMQLLRDNLTLWAS
OsG             ISVFYCEIMNSPD--KACQLAKNAFDEAVAEL-PSLSEENYKDSTLIMQLLRDNLALWNS
Tg1             YSVFFYEILNLPQ--QACEMAKRAFDDAITEF-DNVSEDSYKDSTLIMQLLRDNLTLWTS
Sm4             FSVFYYEILNNSE--QACKCARVAFDSAIAEL-DTLSEESYKDSTIIMQLLRDNLTLWTS
Sp25            FSVFYYEILDSPE--SACHLAKQVFDEAISEL-DSLSEESYKDSTLIMQLLRDNLTLWTS
Sp24            FSVFYYEILNSPD--RACYLAKQAFDEAISEL-DSLSEESYKDSTLIMQLLRDNLTLWTS
```

```
OsA             FSVFYYEILNSPD--RACTLAKQAFDEAISEL-DTLGEESYKDSTLIMQLLRDNLTLWTS
OsD             FSVFYYEILNSPD--RACNLAKQAFDEAISEL-DSLGEESYKDSTLIMQLLRDNLTLWTS
OsF             FSVFYYEILNSPD--RACNLAKQAFDDAIAEL-DTLGEESYKDSTLIMQLLRDNLTLWTS
OsC             FSVFYYEILNSPD--KACNLAKQAFDEAISEL-DTLGEESYKDSTLIMQLLRDNLTLWTS
OsE             FSVFYYEILNSPD--RACNLAKQAFDEAISEL-DTLSEESYKDSTLIMQLLRDNLTLWTS
OsB             FSVFYYEILNSPD--RACNLAKQAFDEAISEL-DTLSEESYKDSTLIMQLLRDNLTLWTS
Ci              FSVFYYEILNSPD--RACRLAKEAFDQAIAEL-DTLSEESYKDSTLIMQLLRDNLTLWTS
DmEP            FSVFYYEILNSPD--RACRLAKAAFDDAIAEL-DTLSEESYKDSTLIMQLLRDNLTLWTS
DrEP2           FSVFYYEILNSPD--RACRLAKAAFDDAIAEL-DTLSEDSYKDSTLIMQLLRDNLTLWTS
XtEP            FSVFYYEILNSPD--RACRLAKAAFDDAIAEL-DTLSEESYKDSTLIMQLLRDNLTLWTS
DrEP            FSVFYYEILNSPD--RACRLAKAAFDDAIAEL-DTLSEESYKDSTLIMQLLRDNLTLWTS
HsEP            FSVFYYEILNSPD--RACRLAKAAFDDAIAEL-DTLSEESYKDSTLIMQLLRDNLTLWTS
MmEP            FSVFYYEILNSPD--RACRLAKAAFDDAIAEL-DTLSEESYKDSTLIMQLLRDNLTLWTS
GgEP            FSVFYYEILNSPD--RACRLAKAAFDDAIAEL-DTLSEESYKDSTLIMQLLRDNLTLWTS
Ca              FSVFYYEILNSPD--RACHLAKQAFDDAVADL-ETLSEDSYKDSTLIMQLLRDNLTLWTD
Sc1             FSVFYYEIQNSPD--KACHLAKQAFDDAIAEL-DTLSEESYKDSTLIMQLLRDNLTLWTS
Sc2             FSVFYYEIQNSPD--KACHLAKQAFDDAIAEL-DTLSEESYKDSTLIMQLLRDNLTLWTS
GgS             FSVFHYEIANAPE--QAISLAKTTFDEAMGDL-HTLSEDSYKDSTLIMQLLRDNLTLWTA
HsS             FSVFHYEIANSPE--EAISLAKTTFDEAMADL-HTLSEDSYKDSTLIMQLLRDNLTLWTA
MmS             FSVFHYEIANSPE--EAISLAKTTFDEAMADL-HTLSEDSYKDSTLIMQLLRDNLTLWTA
DrET            FSVFYYEIQNAPE--QACQLAKEAFDDAIGHL-DNLNEDSYKDSTLIMQLLRDNLTLWTS
XtG             YSVFYYEIQNAPE--QACHLAKTAFDDAIAEL-DTLNEDSYKDSTLIMQLLRDNLTLWTS
HsG             YSVFYYEIQNAPE--QACHLAKTAFDDAIAEL-DTLNEDSYKDSTLIMQLLRDNLTLWTS
MmG             YSVFYYEIQNAPE--QACHLAKTAFDDAIAEL-DTLNEDSYKDSTLIMQLLRDNLTLWTS
GgG             YSVFYYEIQNAPE--QACHLAKTAFDDAIAEL-DTLNEDSYKDSTLIMQLLRDNLTLWTS
DrG             YSVFYYEIQNAPE--QACHLAKTAFDDAIAEL-DTLNEDSYKDSTLIMQLLRDNLTLWTS
DrG1            YSVFYYEIQNAPE--QACHLAKTAFDDAIAEL-DTLNEDSYKDSTLIMQLLRDNLTLWTS
XtET            FSVFYYEIQGNPE--QACLLAKQAFDDAIAEL-DTLNEDSYKDSTLIMQLLRDNLTLWTS
GgET            FSVFYYEIQNAPE--QACLLAKQAFDDAIAEL-DTLNEDSYKDSTLIMQLLRDNLTLWTS
HsET            FSVFYYEIQNAPE--QACLLAKQAFDDAIAEL-DTLNEDSYKDSTLIMQLLRDNLTLWTS
MmET            FSVFYYEIQNAPE--QACLLAKQAFDDAIAEL-DTLNEDSYKDSTLIMQLLRDNLTLWTS
XtT             FSVFYYEILNSPE--KACTLAKNAFDEAIAEL-DTLNEESYKDSTLIMQLLRDNLTLWTS
DrZ             FSVFYYEILNSPE--QACDLAKKAFDDAISEL-DQLTEDSYKDSTLIMQLLRDNLTLWTS
DmZ             FSVFYYEILNSPD--KACQLAKQAFDDAIAEL-DTLNEDSYKDSTLIMQLLRDNLTLWTS
GgT             FSVFYYEILNNPE--LACTLAKTAFDEAIAEL-DTLNEDSYKDSTLIMQLLRDNLTLWTS
HsT             FSVFYYEILNNPE--LACTLAKTAFDEAIAEL-DTLNEDSYKDSTLIMQLLRDNLTLWTS
MmT             FSVFYYEILNNPE--LACTLAKTAFDEAIAEL-DTLNEDSYKDSTLIMQLLRDNLTLWTS
DrT             FSVFYYEILNSPE--QACSLAKQAFDEAIAEL-DTLNEDSYKDSTLIMQLLRDNLTLWTS
DrTB            FSVFFYEILNSPE--KACSLAKQAFDEAIAEL-DTLNEESYKDSTLIMQLLRDNLTLWTS
XtZ             FSVFYYEILNCPE--KACSLAKAAFDEAIAEL-DTLSEESYKDSTLIMQLLRDNLTLWTS
GgZ             FSVFYYEILNSPE--KACSLAKTAFDEAIAEL-DTLSEESYKDSTLIMQLLRDNLTLWTS
HsZ             FSVFYYEILNSPE--KACSLAKTAFDEAIAEL-DTLSEESYKDSTLIMQLLRDNLTLWTS
MmZ             FSVFYYEILNSPE--KACSLAKTAFDEAIAEL-DTLSEESYKDSTLIMQLLRDNLTLWTS
XtA             FSVFYYEILNSPE--KACSLAKSAFDEAIAEL-DTLNEESYKDSTLIMQLLRDNLTLWTS
GgA             FSVFYYEILNSPE--KACNLAKTAFDEAIAEL-DTLNEESYKDSTLIMQLLRDNLTLWTS
HsA             FSVFYYEILNSPE--KACSLAKTAFDEAIAEL-DTLNEESYKDSTLIMQLLRDNLTLWTS
MmA             FSVFYYEILNSPE--KACSLAKTAFDEAIAEL-DTLNEESYKDSTLIMQLLRDNLTLWTS
DrAB            FSVFYYEILNSPE--NACQLAKTAFDEAIAEL-DTLNEDSYKDSTLIMQLLRDNLTLWTS
DrAA            FSVFYYEILNTPE--QACSLAKTAFDEAIAEL-DTLNEDSYKDSTLIMQLLRDNLTLWTS
                 .              *      .:  :   :        :      :.:* ..:  *

Tg3             ESRKD----------------------AMYDWWSSL-------------
Tg2             ETGRTDVQALLGMDYRSLS--------SGQSLDAGSTASFA--------
OsH             ELTGG----------------------ETSKDDDVVMEG----------
Sm1             EREAA----------------------Q---------------------
Sm5             DPERD----------------------DNVKKDTDEKA-----------
Sm2             DQTA-----------------------EGDVENDS--------------
```

```
Sm3     DQTAE--------------------------GDGNDS------------
OsG     DMADDADDIRERT--------------DTTGAKGDPAA-----------
Tg1     DLQADQQQQ------------------EGGEKPAEQADQ----------
Sm4     NSEGE----------------------KDTASPKGDKK-----------
Sp25    DAEYNQSAKEEAPA-------------AAAASENEHPEPKESTTDTVKA
Sp24    DAEYSAAAAGGNT--------------EGAQENAPSNAPEGEAEPKADA
OsA     DMQDDGGDEMR----------------DATKPEDEH-------------
OsD     DANDDGGDEIK----------------EAAAPKEPGDQ-----------
OsF     DNAEDGGDEIK----------------EAAKPEGEGH------------
OsC     DLTEDGGDEVK----------------EASKGDACEGQ-----------
OsE     DISEDAAEEIK----------------EAPKGESGDGQ-----------
OsB     DISEDTAEEIR----------------EAPKRDSSEGQ-----------
Ci      DMQGDA---------------------DGHEGNTDEVQEMHATEAS---
DmEP    DMQAEEV--------------------DPNAGDGEPKEQIQDVEDQDVS
DrEP2   DIQGD----------------------DL--------------------
XtEP    DMQGDGEEQNK----------------DALQDVEDENQ-----------
DrEP    DMQGDGEEQNK----------------EALQDVEDENQ-----------
HsEP    DMQGDGEEQNK----------------EALQDVEDENQ-----------
MmEP    DMQGDGEEQNK----------------EALQDVEDENQ-----------
GgEP    DMQGDGEEQNK----------------EALQDVEDENQ-----------
Ca      LSEAPAATEEQQQSSQAPAA-------QPTEGKADQE------------
Sc1     DMSESGQAEDQQQQQQHQQQQ------PPAAAEGEAPK-----------
Sc2     DISESGQEDQQQQQQQQQQQQQQQQQAPAEQTQGEPTK-----------
GgS     ECAGE----------------------DGGEAGEEPKN-----------
HsS     DNAGE----------------------EGGEAPQEPQS-----------
MmS     DSAGE----------------------EGGEAPEEPQS-----------
DrET    DQQDS----------------------EGGDANN---------------
XtG     DQQDD----------------------DGGEGNN---------------
HsG     DQQDD----------------------DGGEGNN---------------
MmG     DQQDD----------------------DGGEGNN---------------
GgG     DQQDD----------------------DGGEGNN---------------
DrG     DQQDD----------------------EGGEGNN---------------
DrG1    DQQDD----------------------EGGEGNN---------------
XtET    DQQDE----------------------ETGEGNN---------------
GgET    DQQDE----------------------EAGEGNN---------------
HsET    DQQDE----------------------EAGEGN----------------
MmET    DQQDE----------------------EAGEGN----------------
XtT     DTACD----------------------DNDAVEQGENN-----------
DrZ     DNQAD----------------------GDETEEGRQN------------
DmZ     DTQGD----------------------EAEPQEGGDN------------
GgT     DSAGE----------------------ECDAAEGAEN------------
HsT     DSAGE----------------------ECDAAEGAEN------------
MmT     DSAGE----------------------ECDAAEGAEN------------
DrT     DNAAD----------------------EGEGGDGGEN------------
DrTB    DNAPD----------------------EGEGGEGGEN------------
XtZ     DTQGD----------------------EAEQGEGGEN------------
GgZ     DTQGD----------------------EAEAGEGGEN------------
HsZ     DTQGD----------------------EAEAGEGGEN------------
MmZ     DTQGD----------------------EAEAGEGGEN------------
XtA     ENQGE----------------------EADNAEADN-------------
GgA     ENQGD----------------------EGDAGEGEN-------------
HsA     ENQGD----------------------EGDAGEGEN-------------
MmA     ENQGD----------------------EGDAGEGEN-------------
DrAB    ENQGE----------------------EAGENEN---------------
DrAA    ENQGD----------------------EGDAGE-GEN------------
```
