# Supplementary material for: Bioinformatic analysis reveals new determinants of antigenic 14-3-3 proteins and a novel antifungal strategy
Source: PLoS One. 2017 Dec 12;12(12):e0189503. doi: 10.1371/journal.pone.0189503 (PMC5726717; doi:10.1371/journal.pone.0189503)
Supplement: S1 Table — Accession Numbers of All 14-3-3 Proteins. obtained from NCBI, are listed. (PDF) [file pone.0189503.s006.pdf]

<u>**Table 1: Accession Number of 14-3-3 Proteins Analyzed in The Current Study.**</u>

Species	Gene	mRNA	Gene ID	Protein
Homo sapiens	YWHAZ	NM_001135699.1	7534	NP_001129171
Homo sapiens	YWHAQ	NM_006826.3	10971	NP_006817.1
Homo sapiens	SFN	NM_006142.3	2810	NP_006133.1
Homo sapiens	YWHAE	NM_006761.4	7531	NP_006752.1
Homo sapiens	YWHAB	NM_003404.4	7529	NP_003395.1
Homo sapiens	YWHAG	NM_012479.3	7532	NP_036611.2
Homo sapiens	YWHAH	NM_003405.3	7533	NP_003396.1
Mus musculus	Ywhaz	NM_011740.3	22631	NP_035870.1
Mus musculus	Sfn	NM_018754.2	55948	NP_061224.2
Mus musculus	Ywhae	NM_009536.4	22627	NP_033562.3
Mus musculus	Ywhaq	NM_011739.3	22630	NP_035869.1
Mus musculus	Ywhab	NM_018753.6	54401	NP_061223.2
Mus musculus	Ywhag	NM_018871.3	22628	NP_061359.2
Mus musculus	Ywhah	NM_011738.2	22629	NP_035868.1
Gallus gallus	SFN	NM_001293176.1	408037	NP_001280105
Gallus gallus	YWHAZ	NM_001031343.1	425619	NP_001026514.1
Gallus gallus	YWHAE	NM_001006219.1	417554	NP_001006219.1
Gallus gallus	YWHAG	NM_001031477.1	427820	NP_001026648.1
Gallus gallus	YWHAQ	NM_001006415.1	421932	NP_001006415.1
Gallus gallus	YWHAB	NM_001006289.1	419190	NP_001006289.1
Gallus gallus	YWHAH	NM_001007839.1	416955	NP_001007840.1
Xenopus tropicalis	ywhaq	NM_001030399.1	XB-GENE-946182	NP_001025570.1
Xenopus tropicalis	ywhag	NM_001078841.1	XB-GENE-951350	NP_001072309.1
Xenopus tropicalis	ywhah	NM_001016868.2	XB-GENE-949031	NP_001016868.1

Table -1 (Cont'd)

Xenopus tropicalis	ywhab	NM_001011116.1	XB-GENE-1006026	NP_001011116.1
Xenopus tropicalis	ywhae	NM_001008155.1	XB-GENE-996375	NP_001008156.1
Xenopus tropicalis	ywhaz	NM_203842.1	XB-GENE-970616	NP_989173.1
Danio rerio	ywhae1	NM_212605.2	322060	NP_997770.1
Danio rerio	ywhabb	NM_213145.1	406419	NP_998310.1
Danio rerio	ywhaba	NM_001082798.1	323055	NP_001076267.1
Danio rerio	ywhag1	NM_213022.2	117604	NP_998187.1
Danio rerio	ywhaqa	NM_201513.1	399487	NP_958921.1
Danio rerio	ywhaqb	NM_201484.1	335195	NP_958892.1
Danio rerio	ywhah	NM_213164.2	406443	NP_998329.1
Danio rerio	ywhag2	XM_683958.7	560560	XP_689050.2
Danio rerio	ywhae2	NM_001013341.2	503763	NP_001013359.1
Danio rerio	ywhaz	NM_212757.2	336610	NP_997922.2
D. melanogaster	14-3-3zeta	NM_165740.3	FBgn0004907	NP_724884.1
D. melanogaster	14-3-3epsilon	NM_169796.2	FBgn0020238	NP_732309.1
S. cerevisiae	BMH1	NM_001179067.3	856924	NP_011104
S. cerevisiae	BMH2	NM_001180407.3	851676	NP_010384.3
Candida albicans	BMH1	XM_716419.1	3636810	XP_721512.1
T. gondii- tg1	TGME49_063090	XM_002365368.1	7895486	XP_002365409.1
T. gondii- tg3	TGME49_227952	XM_018780187.1	29769078	XP_018636082.1
T. gondii- tg2	TGME49_269960	XM_018781564.1	7895058	XP_018636600.1
S. mansoni	Smp_041430.1	XM_018791040.1	8344048	XP_018645749.1
S. mansoni	Smp_009760	XM_018792041.1	8352517	XP_018644942.1
S. mansoni	Smp_009780.1	XM_018792040.1	8352521	XP_018644940.1
S. mansoni	Smp_002410	XM_018791529.1	8342418	XP_018645303.1
S. mansoni	Smp_034840.1	XM_018793416.1	8344478	XP_018647931.1
C. intestinalis	ci14-3-3	NM_001032441.1	445611	NP_001027613.1

Table -1 (Cont'd)

Oryza sativa Japonica E	LOC4329775	XM_015770740.1	4329775	XP_015626226.1
Oryza sativa Japonica C	LOC4345634	XM_015794813.1	4345634	XP_015650299.1
Oryza sativa Japonica A	LOC4345850	XM_015794321.1	4345850	XP_015649807.1
Oryza sativa Japonica D	LOC4350697	XM_015761572.1	4350697	XP_015617058.1
Oryza sativa Japonica B	LOC4336066	XM_015780240.1	4336066	XP_015635726.1
Oryza sativa Japonica F	LOC4333880	XM_015775647.1	4333880	XP_015631133.1
Oryza sativa Japonica G	LOC4325534	XM_015773761.1	4325534	XP_015629247.1
Oryza sativa Japonica H	LOC9269633	XM_015760076	9269633	XP_015615562
Schizosaccharomyces pombe	rad24	NM_001019591.2	2542029	NP_594167.1
Schizosaccharomyces pombe	rad25	NM_001019670.2	2542252	NP_594247.1
