# Supplementary material for: Bioinformatic analysis reveals new determinants of antigenic 14-3-3 proteins and a novel antifungal strategy
Source: PLoS One. 2017 Dec 12;12(12):e0189503. doi: 10.1371/journal.pone.0189503 (PMC5726717; doi:10.1371/journal.pone.0189503)
Supplement: S2 Table — Position of 14-3-3 isoforms In different species is listed. (PDF) [file pone.0189503.s007.pdf]

Table 2: Chromosomal Location of 14-3-3 Proteins in Different Species

	Species						
**Isoform**	**Human**	**Rat**	**Mouse**	**Xenopus**	**Drosophila**	**Chicken**	**Zebrafish**
YWHAB	20	3	2	10		20	6,8
YWHAZ	8	7	15	6	2R	2	19
YWHAE	17	10	11	2	3R	19	7,15
YWHAH	22	14	5	1		15	10
YWHAQ	2	6	12	5		3	20,17
YWHAG	7	12	5	2		19	5,15
SFN	1	5	4	N/A		23	N/A

Species	Isoform	Chromosome Number
**Toxoplasma 14-3-3**		
	Tg1	VIIb
	Tg2	VIII
	Tg3	X
S. cerevisiae		
	BMH1	V
	BMH2	IV
C. albicans		
	BMH	1
S. pombe		
	Rad24	I
	Rad25	I
